# Loss of *Hif-2α* Rescues the *Hif-1α* Deletion Phenotype of Neonatal Respiratory Distress In Mice

**DOI:** 10.1371/journal.pone.0139270

**Published:** 2015-09-30

**Authors:** Yogesh Saini, Steven P. Proper, Peter Dornbos, Krista K. Greenwood, Anna K. Kopec, Scott G. Lynn, Elizabeth Grier, Lyle D. Burgoon, Timothy R. Zacharewski, Russell S. Thomas, Jack R. Harkema, John J. LaPres

**Affiliations:** 1 Department of Biochemistry and Molecular Biology, Michigan State University, East Lansing, Michigan, United States of America; 2 Genetics Program, Michigan State University, East Lansing, Michigan, United States of America; 3 Center for Integrative Toxicology, Michigan State University, East Lansing, Michigan, United States of America; 4 College of Osteopathic Medicine, Michigan State University, East Lansing, Michigan, United States of America; 5 The Hamner Institutes for Health Sciences, Research Triangle Park, North Carolina, United States of America; 6 Department of Pathobiology and Diagnostic Investigation, Michigan State University, East Lansing, Michigan, United States of America; 7 Center for Mitochondrial Science and Medicine, Michigan State University, East Lansing, Michigan, United States of America; University of Tennessee Health Science Center, UNITED STATES

## Abstract

Hypoxia is a state of decreased oxygen reaching the tissues of the body. During prenatal development, the fetus experiences localized occurrences of hypoxia that are essential for proper organogenesis and survival. The response to decreased oxygen availability is primarily regulated by hypoxia-inducible factors (HIFs), a family of transcription factors that modulate the expression of key genes involved in glycolysis, angiogenesis, and erythropoiesis. HIF-1α and HIF-2α, two key isoforms, are important in embryonic development, and likely are involved in lung morphogenesis. We have recently shown that the inducible loss of *Hif-1α* in lung epithelium starting at E4.5 leads to death within an hour of parturition, with symptoms similar to neonatal respiratory distress syndrome (RDS). In addition to *Hif-1α*, *Hif-2α* is also expressed in the developing lung, although the overlapping roles of *Hif-1α* and *Hif-2α* in this context are not fully understood. To further investigate the independent role of *Hif-2α* in lung epithelium and its ability to alter *Hif-1α*-mediated lung maturation, we generated two additional lung-specific inducible *Hif-α* knockout models (*Hif-2α* and *Hif-1α+Hif-2α*). The intrauterine loss of *Hif-2α* in the lungs does not lead to decreased viability or observable phenotypic changes in the lung. More interestingly, survivability observed after the loss of both *Hif-1α* and *Hif-2α* suggests that the loss of *Hif-2α* is capable of rescuing the neonatal RDS phenotype seen in *Hif-1α*-deficient pups. Microarray analyses of lung tissue from these three genotypes identified several factors, such as *Scd1*, *Retlnγ*, and *Il-1r2*, which are differentially regulated by the two HIF-α isoforms. Moreover, network analysis suggests that modulation of hormone-mediated, NF-κB, C/EBPα, and c-MYC signaling are central to HIF-mediated changes in lung development.

## Introduction

Developmental defects specific to the lung fail to manifest in utero, when gas and nutrient exchange are performed via the utero-placental interface. However, proper intrauterine development of the alveolar gas exchange regions of the lung is essential for the newborn’s first breath and sustained life outside the womb [1]. Similar to other tissues in the fetus, lung development is a highly coordinated process involving complex intracellular and extracellular signals that control transcriptional programs leading to proper cellular behavior and morphogenesis. Lung morphogenesis and alveolarization are regulated through the temporo-spatial expression of a plethora of transcription factors, growth regulators, and environmental signals [2–5].

One important extracellular signal for proper lung development is oxygen availability, and a decrease in available oxygen, referred to as hypoxia, is an essential component during proper fetal development [6]. Sensing hypoxia is critical for metabolic homeostasis as well as proper development. Mammals use a family of proteins called hypoxia inducible factors (HIFs) to perform this sensing task. There are three cytosolic HIFs: HIF-1α, HIF-2α, and HIF-3α; each is oxygen labile. In the presence of adequate oxygen, these HIFs are modified by prolyl hydroxylase domain-containing proteins (PHDs), in an oxygen-, iron(II)- and α-ketoglutarate-dependent manner. Upon hydroxylation, the cytosolic HIF-α subunit becomes a substrate for ubiquitination by the Von Hippel Lindau (VHL) tumor suppressor protein and, thereafter, the HIF-α is degraded via the proteasome. Under the conditions of oxygen deprivation, PHD activity is inhibited leading to the stabilization and translocation of HIF-α into the nucleus and subsequent dimerization with the aryl hydrocarbon nuclear translocator (ARNT, also known as HIF-1β). The active dimer binds to the hypoxia response element (HRE) sequence in the promoter region of hypoxia-responsive genes (for review, see [7, 8]).

Proteins encoded by HIF target genes play important roles in glycolysis, angiogenesis, erythropoiesis, and development. In the developing fetus, low oxygen levels allow activation of HIF-regulated pathways needed for proper organogenesis [9]. The importance of HIF signaling in embryonic development is best illustrated in homozygous null mouse models of *Hif-1α*, *Hif-2α*, or *Arnt*, which all show embryonic lethality by approximately E10.5 [9–11]. Mice lacking *Hif-1α*, the most ubiquitously expressed *Hif*, display disorganized yolk sac/neural fold/cephalad vascularization, lack of neural tube closure, and an array of cardiovascular defects [12–14]. *Hif-2α* knockout mice displayed a wider variety of phenotypes, thought to be strain-dependent, including bradycardia from reduced catecholamine synthesis, yolk sac vascular defects similar to *Hif-1α* knockouts, impaired lung maturation, and multiple organ malformations [15–18]. Mice lacking *Arnt* (*Hif-1β*) also display similar embryonic lethal phenotypes and further illustrate the importance of HIF signaling in placental formation [19–23]. The mid-gestational lethality of the systemic knockout models has made it challenging to study HIFs during fetal lung development.

Both HIF-1α and HIF-2α protein are expressed in the developing lung, although their distinct spatial and temporal expression patterns suggest differential roles in lung maturation. In first trimester human lungs, HIF-1α is found prominently in branching epithelium, while HIF-2α is found both in epithelial cells as well as mesenchymal tissues likely to form vasculature [24, 25]. In mouse lungs, *Hif-2α* expression increases dramatically prior to parturition and remains highly expressed through adulthood. Adult lungs contain higher levels of *Hif-2α* mRNA than any other organ under normoxic conditions [3, 26, 27]. HIF-2α protein is easily detectable in embryonic mouse lungs, where it is found in the nuclei of bronchial and alveolar epithelial cells and interstitial cells [28]. As the lung matures, HIF-2α protein is found less in parenchyma and primarily in the bronchial and bronchiolar epithelial cells [28]. *Hif-1α* is more highly expressed in early lung development and slowly decreases expression as the lung matures [27]. HIF-1α protein is found primarily in epithelial cells in early lung development and as the lungs mature can be found in bronchial and alveolar Type II epithelial cells [27, 28]. Recent work has illustrated the vital role that HIFs play in post-natal lung development, especially in relation to vascular development and alveolar growth in hyperoxic injury [29].

Tissue-specific knockout/induction models have shown that *Hif-1α* must be tightly regulated in order to achieve proper lung development. We have previously shown that in utero Cre-mediated recombination of *Hif-1α* in cells expressing surfactant protein C (*Sp-c*) led to neonatal respiratory distress syndrome (RDS) in mice, with impaired surfactant production, excess glycogen storage in parenchymal epithelial cells, and impaired cellular differentiation [30]. Bridges et al. used the *Sp-c*-driven induction of two oxygen-stable HIF-1α constructs, which both showed neonatal RDS at birth, with varying effects to vascular components and branching morphogenesis in the lung. Tibboelet al. had created mice with constitutively active *Hif-1α* (*Hif-1αΔODD*) driven by the *Sp-c* promoter, which, in contrast to the stable HIF-1α-constructs of Bridges *et al*., caused increased vascularization and alveolarization [31]. Differences in these stable *Hif-1α* mutants are thought to be due to the differences in how these constructs were created. A similar method inducing stable *Hif-2α* expression in the *Sp-c* model was employed by Huang et al. that also demonstrated neonatal RDS upon parturition, increased glycogen stores, and aberrant Type II cells [32]. Regardless of the specific reasons that these similar models caused such different outcomes, these differences highlight how tightly regulated HIF-signaling must be during lung development to promote normal functioning and proper structural development.

While these studies certainly solidify the importance of HIFs in lung development, the collective role of HIF-1α and HIF-2α in lung development, their place in the transcriptional network necessary for proper cellular differentiation, and their potential redundant function in lung development are still unclear. To further enhance our understanding of the role of these two HIFs in lung development, mice with lung-specific deletions of *Hif-1α* (termed *Hif-1αΔ/Δ*), *Hif-2α* (termed *Hif-2αΔ/Δ*), and both *Hif-1α* and *Hif-2α* (termed *Hif-1/2αΔ/Δ*) were generated. These studies confirmed that HIF-1α is essential for proper lung development. Interestingly, the lung specific deletion of *Hif-2α* led to no deleterious phenotype and displayed no lethality upon parturition. More importantly, simultaneous deletion of both isoforms of HIF rescued the respiratory distressed phenotype that was observed after *Hif-1α* deletion. In complementary microarray studies, a battery of genes was identified consistent with the observed phenotypes including factors involved in lung development and function such as *Il1r2*, *Retlnγ*, and *C/ebp*α. These results demonstrate a non-redundant function for HIF-1α and HIF-2α in lung development and identify hypoxia and HIF-mediated signaling pathways that are altered upon their manipulation.

## Material and Methods

### Transgenic mice and genotyping

*Hif-1α* ^*flox/flox*^, *Hif-2α* ^*flox/flox*^ and *SPC-rtTA* ^*-/tg*^ */ (tetO)*_*7*_ *-CMV-Cre* ^*tg/tg*^ transgenic mice were generous gifts from Randall Johnson (University of Cambridge), M. Celeste Simon (University of Pennsylvania) and Jeffrey Whitsett (Cincinnati Children’s Hospital Medical Center), respectively [12, 33–36]. In the present study, we used these transgenic mice to generate three genotypically different mouse lines:

$$SPC-rtTA^{-/tg}/{\left(tetO\right)}_{7}-CMV-Cre^{tg/tg}/Hif-1a^{flox/flox},$$

$$SPC-rtTA^{-/tg}/{\left(tetO\right)}^{7}-CMV-Cre^{tg/tg}/Hif-2a^{flox/flox},$$

$$SPC-rtTA^{-/tg}/{\left(tetO\right)}_{7}-CMV-Cre^{tg/tg}/Hif-1a^{flox/flox}/Hif-2a^{flox/flox}$$

[30].

**Fig 1:**
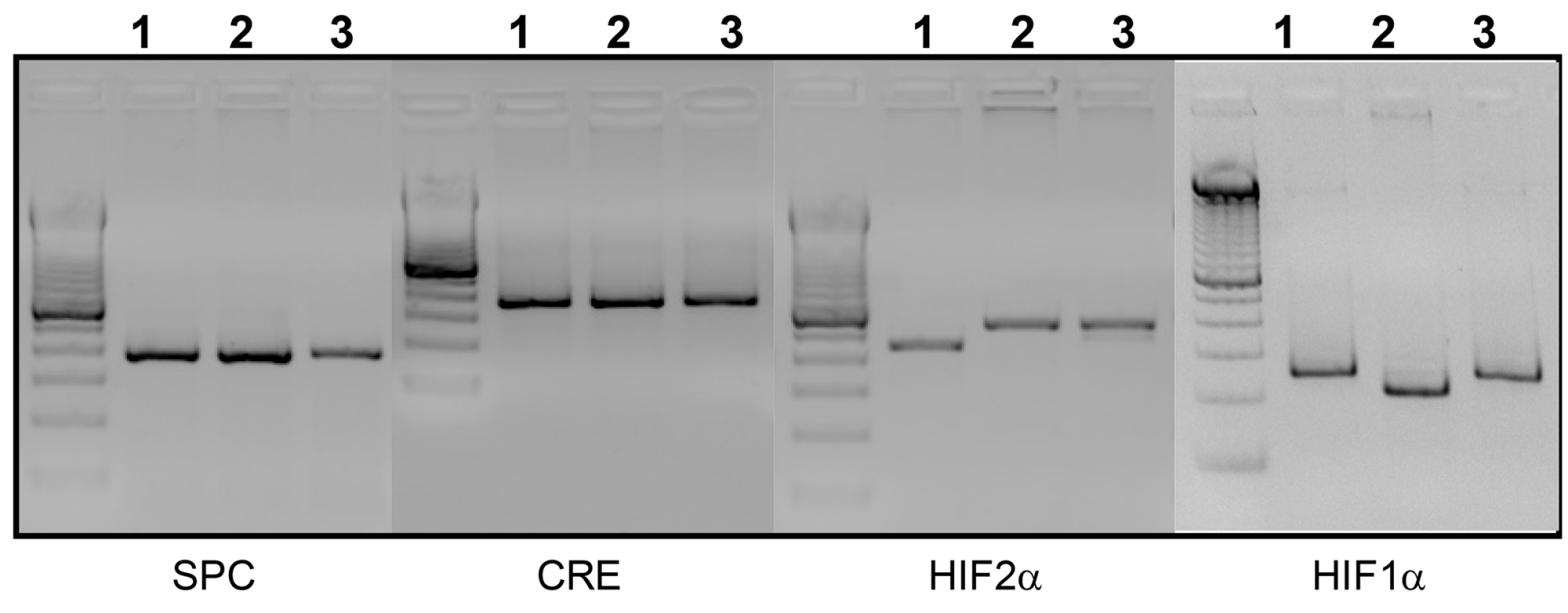
Genotyping of transgenes. PCR genotyping was performed for all four transgenes as described in materials and methods. Sizes of the amplified products obtained are: 240 bp for *Hif-1α* (wild type); 274 bp for *Hif-1α* ^*flox/flox*^; 410 bp for *Hif-2α* (wild type); 444bp for *Hif-2α* ^*flox/flox*^; 370 bpfor Cre transgene; 350 bpfor rtTA transgene. One representative sample was genotyped for the four transgenes from each of three generated mouse strains: *SPC-rtTA* ^*-/tg*^ */(tetO)*_*7*_ *-Cre* ^*-/tg*^ */Hif-1α* ^*fl/fl*^ (**Lane 1**), *SPC-rtTA* ^*-/tg*^ */(tetO)*_*7*_ *-Cre* ^*-/tg*^ */Hif-2α* ^*fl/fl*^ mouse (**Lane 2**), and *SPC-rtTA* ^*-/tg*^ */(tetO)*_*7*_ *-Cre* ^*-/tg*^ */Hif-1α/2α* ^*fl/fl*^ (**Lane 3**)).

All three lines are capable of respiratory epithelium-specific conditional recombination in the floxed *Hif-α* alleles. In this model doxycycline was administered to the dams and, depending on the day, various cell populations within the lung underwent recombination of the floxed alleles [35]. Genotyping of the progeny was performed by PCR for all three loci using previously published primer sequences [30, 33]. Genomic DNA extraction from tail clipping was performed using DirectPCR extraction system (Viagen Biotech, CA) using the manufacturer’s protocol. PCR conditions were standardized for all three alleles: denaturation at 94°C for 3 min; 38 cycles of denaturation at 94°C for 45 sec, annealing at 60°C for 45 sec and polymerization at 72°C for 60 sec followed by a 7 min extension at 72°C. Sizes of the amplified products were 210 bp for Hif-1α (wild type); 244 bp for Hif-1α^flox/flox^; 410 bp for Hif-2α (wild type); 444bp for Hif-2α^flox/flox^; 370 bp for the Cre transgene; 350 bp for the rtTA transgene (Fig 1). Each mouse line was maintained in a mixed C57BL/6:FVB background.

### Doxycycline treatment and animal husbandry

Triple and quadruple transgenic pups were exposed to doxycycline in utero to generate lung epithelium-specific *Hif*-deficient pups. Dams were maintained on the doxycycline feed (625 mg doxycycline/kg; Harlan Teklad, Madison, WI) and drinking water (0.8 mg/ml: MP Biomedicals, Solon, OH) from day embryonic day 4.5 (E4.5) until postnatal day 1 (PN1). It should be noted that this dosing regimen for DOXY is less than is needed for off-target effects and shows no toxicity in the *SPC-rtTA* ^*-/tg*^ */(tetO)*_*7*_ *-Cre* ^*-/tg*^ */Hif-1α* ^*fl/fl*^ model [30, 37]. All *Hif*-deficient pups born from these dams are referred to as *Hif-1αΔ/Δ*, *Hif-2αΔ/Δ* or *Hif-1/2αΔ/Δ*. Triple and quadruple transgenic mice that were not exposed to doxycycline are referred to as controls. All animal handling and necropsy protocols were approved by the Institutional Animal Care and Use Committee (IACUC) at Michigan State University. Mice used in this study were kept at the animal housing facility under the strict hygienic and pathogen-free conditions approved by the University Laboratory Animal Resource (ULAR) regulatory unit.

### Lungs harvesting and processing

Control and *Hif-1αΔ/Δ*, *Hif-2αΔ/Δ* or *Hif-1/2αΔ/Δ* pups were sacrificed within one hour of parturition. All neonatal pups were anesthetized with sodium pentobarbital (50 mg/ml) and after anesthesia was achieved, a midline laparotomy was performed. Pups were sacrificed by exsanguination of the inferior vena cava and the lung and heart were removed en bloc. For histopathological analysis, the lungs were fixed with 10% neutral buffered formalin (Fisher Chemicals, TX).For RNA/gene expression analysis, lungs were stored in RNAlater (Ambion, Austin, TX) at 4°C prior to RNA isolation.

### Histopathology and immunohistochemistry

At least four to six pups of each genotype and doxycycline treatment were histopathologically analyzed. Lung tissue, once isolated, was not inflated but floated in neutral-buffered formalin (10%). Formalin fixed specimens were processed, embedded in paraffin and sectioned on a rotary microtome at 4 μm thickness, deparaffinized in xylene and hydrated through descending grades of ethanol to distilled water. Slides were either stained with hematoxylin and eosin (H&E), periodic acid Schiff (PAS) or immunostained. For immunostaining of HIF-1α and HIF-2α, endogenous peroxidase activity was quenched by incubation with 6% H_2_O_2_ for 30 min. Novus Biologicals (Littleton, CO, USA) Polyclonal Rabbit Hif-1α (NB100-479) diluted 1:250 in PBS and Polyclonal Rabbit Hif-2α (NB100-122) Dilution 1:150 were incubated for 1 hour at RT. Biotinylated anti-Rabbit secondary antibody and streptavidin-bound peroxidase from the Rabbit Vectastain Elite ABC kit (Vector Laboratories, Burlington, CA) were used, according to the manufacturer’s recommendations. For immunostaining of SCD1, Santa Cruz (Paso Robles, CA, USA) Goat Polyclonal (sc-14719, S-15) diluted 1:250 in PBS was incubated for 1 hour at RT. Binding of secondary antibody with streptavidin/biotin paired peroxidase were performed with Goat Vectastain Elite ABC Kit (PK-6105, Vector Laboratories, CA) according to the manufacturer’s recommendations. Reaction for HIF-1α, HIF-2α and SCD1 were developed with Vector DAB peroxidase substrate kit (SK-4100, Vector Laboratories, CA). Slides were then counterstained in Harris’ Hematoxylin for 10 seconds and rinsed in tap water. Slides were then dehydrated through ascending grades of ethanol; cleared with Xylene and cover-slipped using Permount mounting media (SP15, Fisher Scientific, USA).

### Morphometric Analyses of Alveolar Septal Thickness

Lung tissue sections were digitized at a magnification of 20xwith a slide scanner (VS110, Olympus America, Center Valley, PA). newCAST software (VisioPharm, Hoersholm, Denmark) was used for all morphometric analyses of randomly selected digitized profiles of the pulmonary parenchyma. Alveolar septal thickness was determined as previously described [38]. Statistical analyses in comparing the differing groups (n ≥ 4) were made with an ANOVA and a Tukey’s post-hoc in R [39].

### RNA Isolation

Lung tissue (10 mg) stored in RNAlater (Ambion, Life Technologies, USA) was homogenized in RLT buffer (RNeasy RNA isolation Kit, Qiagen, Valencia, CA) using a Mixer Mill 300 tissue homogenizer (Retsch, Haan, Germany).Total RNA quantification was performed spectrophotometrically (NanoDrop ND-1000 UV-Vis Spectrophotometer). Isolated RNA was resuspended in RNAase-free water, quantified (A260), and concentration was calculated by spectrophotometric methods (A260).Purity was assessed by the A260:A280 ratio and by visual inspection of 3 μg on a denaturing gel.

### Microarray Experimental Design

The lung RNA samples extracted from *Hif-1αΔ/Δ*, *Hif-2αΔ/Δ* or *Hif-1/2αΔ/Δ* and control animals were individually hybridized to 4 x 44K whole mouse genome oligo microarrays (Agilent Technologies, Inc., Santa Clara, CA, USA). Hybridizations were performed according to the manufacturer’s protocol (Agilent Manual: G4140-90040 v. 5.7) with four biological replicates per group (non-pooled samples were isolated from different litters) using one-color labeling (Cy3). Briefly, 1 μg of total RNA from each sample was reverse-transcribed to cDNA in the presence of RNA One-Color Spike-In mix, T7 Promoter primer, 5X First Strand Buffer, 0.1M DDT, 10 mMdNTP mix, MLV-RT and RNaseInh during 2 h incubation at 40°C. The cDNA was then used to synthesize fluorescently-labeled cRNA in a second 2 hour incubation at 40°C using 4X Transcription Buffer, 0.1M DDT, NTP mix, 50% PEG, RNaseInh, Inorganic pyrophosphatase, T7 RNA Polymerase and Cyanine 3-CTP (Cy3). The cRNA was purified using RNeasy Isolation Kit (Qiagen) and eluted with RNase-free water. Purified cRNA was assessed for Cy3 absorbance and concentration using NanoDrop spectrophotometry. Fluorescently labeled cRNA was fragmented using 25X Fragmentation Buffer and 10X Blocking Agent for 30 min at 60°C. The fragmentation was stopped by adding 2x GEx Hybridization Buffer HI-RPM and the samples were hybridized for 17 h at 65°C, washed, and scanned at 532 nm (Cy3) on a GenePix 4000B scanner (Molecular Devices, Union City, CA, USA). The microarray images were analyzed for feature and background intensities using GenePix Pro 6.0 (Molecular Devices). All data were managed in TIMS dbZach data management system [40].

### Microarray Analysis and Functional Annotation

All microarray data in this study passed quality assurance protocols [41]. Microarray data were first normalized using a semi-parametric approach, as published previously[42]. Statistical analysis to identify significant differentially expressed genes was performed using empirical Bayes method which uses a mixed ANOVA model for the estimation of model-based t-values to calculate posterior probabilities (P1(*t*) values) of gene activity on a per gene and group basis [43]. Gene expression data were ranked and prioritized using a |fold change| ≥ 1.5 and a statistical P1(*t*) value ≥ 0.95 criteria to identify significant differentially expressed genes. The differentially expressed genes were analyzed using the GeneGoMetaCore database (GeneGo, Inc., St. Joseph, MI). Enrichment analysis was performed for the GeneGo canonical signaling pathways, the gene ontology biological process (GOBP) categories, and the protein functional classes. The protein functional classes represented the standard grouping of pharmacological targets and included transcription factors, receptors, ligands, kinases, proteases, phosphatases, enzymes, and other. The enrichment p-values were calculated based on a hypergeometric distribution with a false discovery rate (FDR) correction. The genes showing differential expression only in the *Hif-1αΔ/Δ* genotype were used as input for constructing GeneGo signaling networks. The networks were constructed based on known physical, biochemical, and molecular interactions that were manually curated from the literature. The transcription regulation analysis was performed using the default settings with a lung expression pre-filter to remove those genes not expressed in the lung. Topology analysis was performed on the networks to assess the extent and type of connectivity within each network. The degree of network connectivity is the average number of edges (interactions) connected to a specific node (gene or protein). The clustering coefficient captures the degree of connectivity between a node’s neighbors. The clustering coefficient is defined as:

$$C_{i}=\frac{2n_{i}}{k_{i}\left(k_{i}-1\right)}$$

where n_i_ is the number of edges among the k_i_ neighbors of node i. As k_i_(k_i_-1)/2 is the maximum number of edges, the clustering coefficient is a number between 0 and 1. The average clustering coefficient is obtained by averaging the clustering coefficient of individual nodes. A high clustering coefficient is characterized by highly connected sub-graphs.

### Real-time PCR analysis

RNA was isolated from five different lungs from mice of different litters and was purified and quantitated as described above. Total RNA (1 μg) was reverse transcribed using superscript II reverse transcriptase kit (Invitrogen, Carlsbad, CA, USA). The expression level of selected genes involved in surfactant metabolism and lung development were analyzed by Real-Time PCR using SYBR green (Applied Biosystems PRISM 7000 Sequence Detection System, Foster City, CA, USA) as previously described [44]. Gene specific primers are listed in S1 Table. Copy number was determined by comparison with standard curves of the respective genes. This measurement was controlled for RNA quality, quantity, and RT efficiency by normalizing it to the expression level of the hypoxanthine guanine phosphoribosyl transferase (*Hprt*) gene. Statistical significance was determined by use of normalized relative changes and an ANOVA and a Tukey’s post-hoc (GraphPad Prism 4 software, La Jolla, CA, USA).

## Results

### Generation of *Hif-1α* and *Hif-2α* deficient mice and their survivability

**Fig 2:**
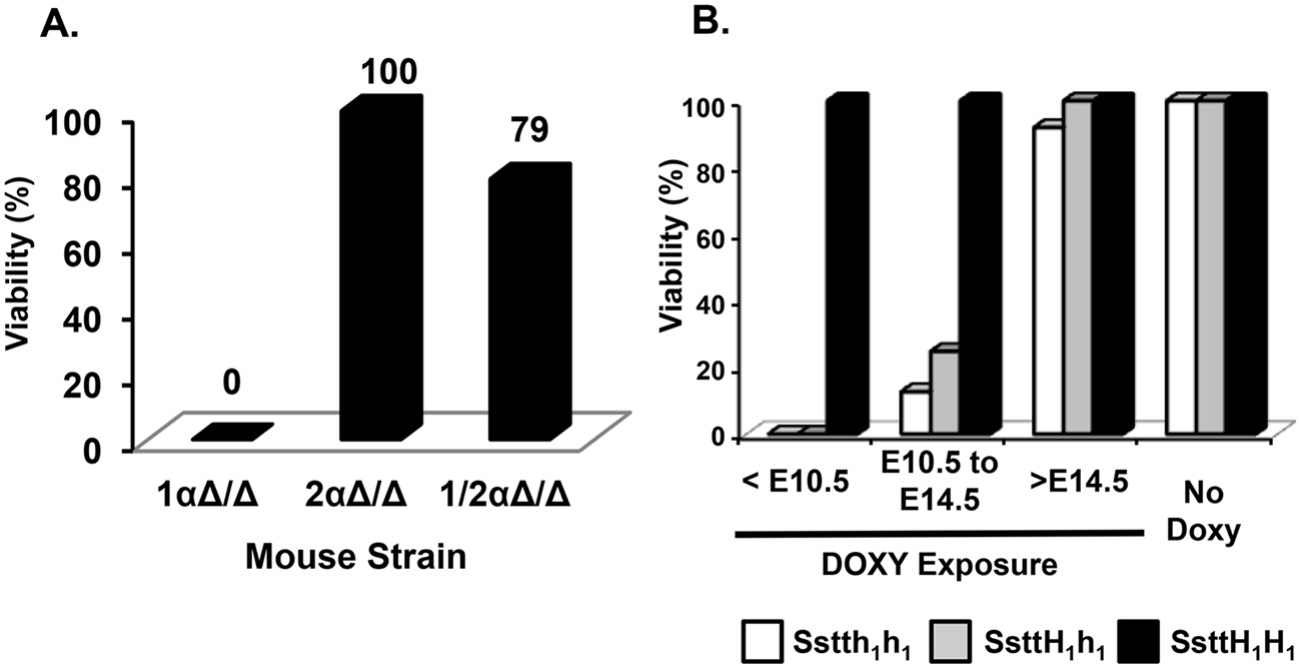
Survivability Plot. Viability of neonatal *Hif-1αΔ/Δ*, *Hif-2αΔ/Δ* and *Hif-1/2αΔ/Δ* pups. (**A**) All *Hif-1αΔ/Δ* pups showed signs of respiratory distress whereas all *Hif-2αΔ/Δ* pups were viable with no obvious phenotype. Simultaneous removal of both *Hifα* forms resulted in approximately 79% survival. (**B**) Time-course of various *Hif-1α* mice with doxycycline (DOXY) exposure <E10.5, E10.5-E14.5 and >E14.5 compared with No DOXY. All mice had *SPC-rtTA* ^*-/tg*^ */(TetO)*_*7*_ *-CMV-Cre* ^*tg/tg*^; white bars indicate homozygous floxed *Hif-1α* (h1h1), grey bars indicate heterozygous floxed *Hif-1α* (H1h1), and black bars indicate wt*Hif-1α* (H1H1).

Three strains of mice were generated that were capable of inducible lung-specific deletion of *Hif-1α*, *Hif-2α*, or *Hif-1α* and *Hif-2α* as described in materials and methods. To achieve recombination of the floxed alleles, dams were exposed to doxycycline as previously described [30]. Dams of genotype controls for the triple or quadruple transgenic embryos were maintained on regular feed and water. In total, 332 *Hif-1αΔ/Δ*, 114 *Hif-2αΔ/Δ*, and 58 of *Hif-1/2αΔ/Δ* pups were observed. All 332 *Hif-1αΔ/Δ* pups exhibited lethality upon parturition from a pathology that resembles RDS (Fig 2A). In contrast, all 114 *Hif-2αΔ/Δ* pups survived with no gross phenotypic consequences of the developmental loss. *Hif-2αΔ/Δ* mice were born in appropriate Mendelian ratios and display no signs of RDS (Fig 2A). These results suggest that developmental removal of *Hif-2α* from the respiratory epithelium is not detrimental to normal development. Interestingly, the quadruple transgenic *Hif-1/2αΔ/Δ* mice also displayed little pathology. Out of observed 58 *Hif-1/2αΔ/Δ* pups, only 12 pups (21%) exhibited RDS symptoms. The remaining 46 double-deficient pups appeared identical to their littermate and genotype controls (Fig 2A). These results suggest that loss of *Hif-2α* can rescue the RDS-like phenotype seen in the *Hif-1αΔ/Δ* mice. For *Hif-1α* mice, a subset of litters were given DOXY at three different time periods, <E10.5, E10.5-E14.5, and >E14.5, and impact of heterozygosity of *Hif-1α* on viability was assessed. We show that mice containing only one functional copy of *Hif-1α* still have very low viability in the earlier developmental time points and that heterozygosity in these mice does not necessarily rescue the neonatal respiratory distress syndrome phenotype for *Hif-1α* (Fig 2B). Finally, mice with no copies of the *Hif-1α* floxed allele exhibited 100% viability at every DOXY exposure suggesting that the lethality was due to recombination of *Hif-1α* and was not due to DOXY-induced off target effects.

### Histopathological analysis of neonatal *Hif*-deficient mice

**Fig 3:**
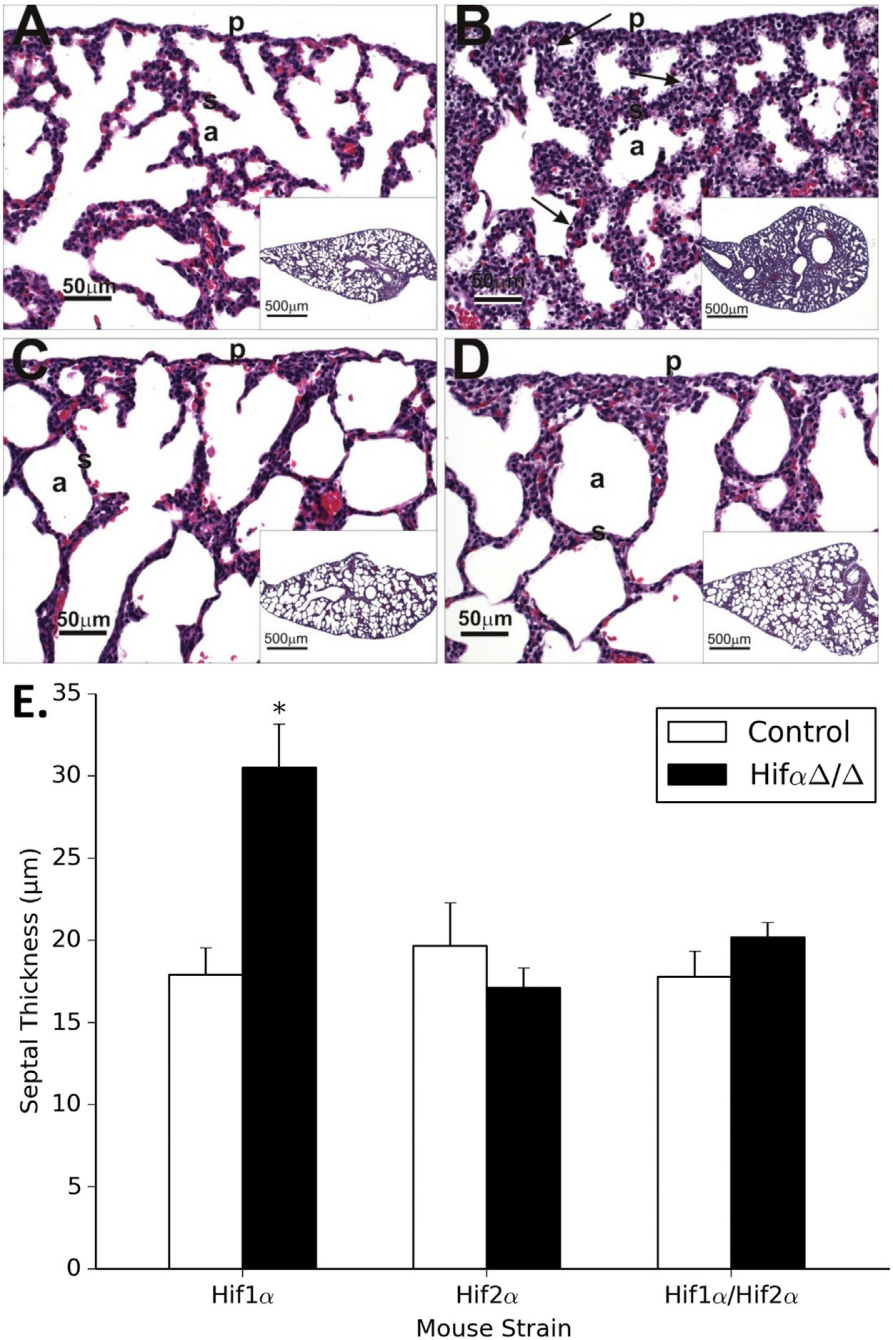
Pulmonary Histopathology. Light photomicrographs of hematoxylin and eosin stained lung sections from control (**A**), *Hif-1αΔ/Δ* (**B**), *Hif-2αΔ/Δ* (**C**) and *Hif-1/2αΔ/Δ* (**D**) pups. Analysis of septal thickness of control mice (**white bars**) and *Hif-1αΔ/Δ* mice (**black bars**) was measured morphometrically as described in materials and methods (**E**). Results are presented as mean alveolar thickness with error bars representing the standard error. Alveolar airspace labeled as (a), septae labeled as (S). * = P ≤ 0.05.

Pups were euthanized approximately 1 hour post-parturition and the lungs were examined. *Hif-1αΔ/Δ* mice confirmed previous finding and displayed attenuated alveolar space and thickened septa (Fig 3B) compared to controls (Fig 3A). In contrast, lungs from *Hif-2αΔ/Δ* mice displayed slightly thinner septa and larger alveolar space (Fig 3C) compared to controls. Finally, the *Hif-1/2αΔ/Δ* pups appeared similar to control pups and displayed normal septa and alveolar architecture (Fig 3D). The change in septal thickness was quantitated morphometrically. The increased septal thickness in the *Hif-1αΔ/Δ* lungs was significantly different as compared to littermate control (p<0.05). There was a slight decrease in septal thickness that was not significantly different in the *Hif-2αΔ/Δ* lungs as compared to their littermate controls (Fig 3E).

### Expression of *Hif-1α* and *Hif-2α* in the three genotypes

**Fig 4:**
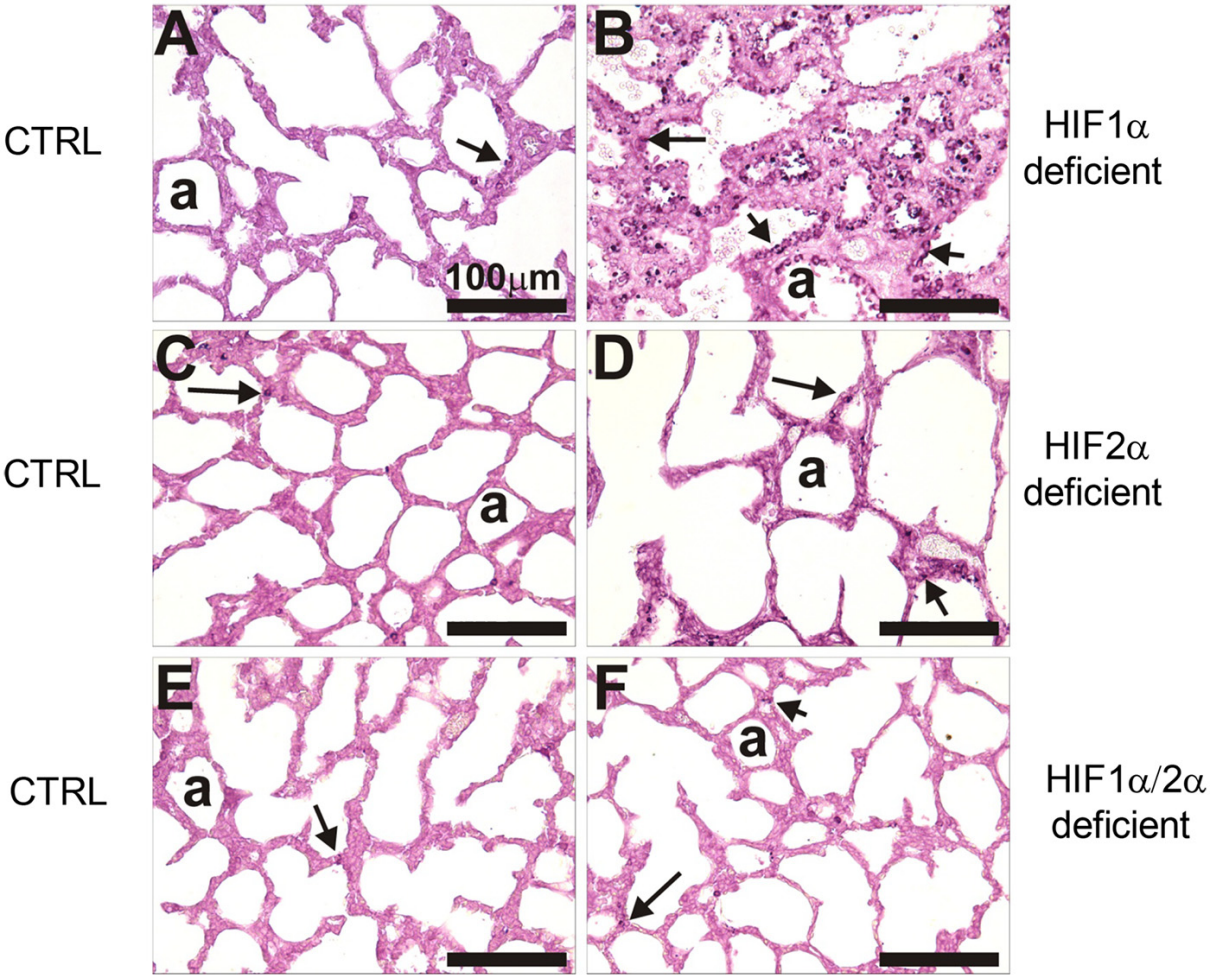
Periodic acid Schiff (PAS) staining. Periodic acid Schiff (PAS) stained lung sections from control (**A, C, E**) and *Hif-1αΔ/Δ* (**B**), *Hif-2αΔ/Δ* (**D**), *Hif-1/2αΔ/Δ* (**F**) pups. Cuboidal epithelium lining alveolar septa in *Hif-1αΔ/Δ* pups (**B**) has greater PAS-stained glycogen (arrows) compared to that of the control pup.

To assess HIF deletion, immunohistochemistry was performed on lung sections from DOXY-treated *Hif-1α* ^*fl/fl*^, *Hif-2α* ^*fl/fl*^, *Hif-1/2α* ^*fl/fl*^ pups, and their respective No-DOXY controls. In neonates, the expression of *Hif-1α* was predominantly localized to alveolar and bronchiolar epithelial cells in controls and *Hif-2αΔ/Δ* mice (S1 Fig, panels A, C, D and E). The level of HIF-1α positive staining was reduced in the *Hif-1αΔ/Δ* and *Hif-1/2αΔ/Δ* mice (S1 Fig, panels B and F). The extent of HIF-1α staining in *Hif-2αΔ/Δ* pups remained unchanged or increased at this time point (S1 Fig, panel D). In neonates, the level of HIF-2α staining was attenuated in each of the three deficient genotypes (S2 Fig, panels B, D and F) as compared to controls (S2 Fig, panels A, C and E) confirming the recombination at this *Hif-2α* floxed locus in the *Hif-2αΔ/Δ* and *Hif-1/2αΔ/Δ* mice. Moreover, it supports the previous observation that loss of *Hif-1α* leads to a decrease in*Hif-2α* expression [30]. The results confirm the functional deletion of the floxed loci and suggest that some level of coordination between the expression of these two hypoxia regulated factors exists and that it plays a role in lung development. During development, the newly differentiated Type II cells use glycogen stores to produce surfactants. Lung sections from *Hif-1αΔ/Δ* pups (Fig 4B) had larger reserves of glycogen as compared to *Hif-2αΔ/Δ* and *Hif-1/2αΔ/Δ* pups (Fig 4D and 4F). PAS-positive cells were absent from the *Hif-2αΔ/Δ* and *Hif-1/2αΔ/Δ* pups and were indistinguishable from control pups (Fig 4A, 4C and 4E).

### Transcriptional Response to *Hif-1α* and *Hif-2α* Deletions

**Fig 5:**
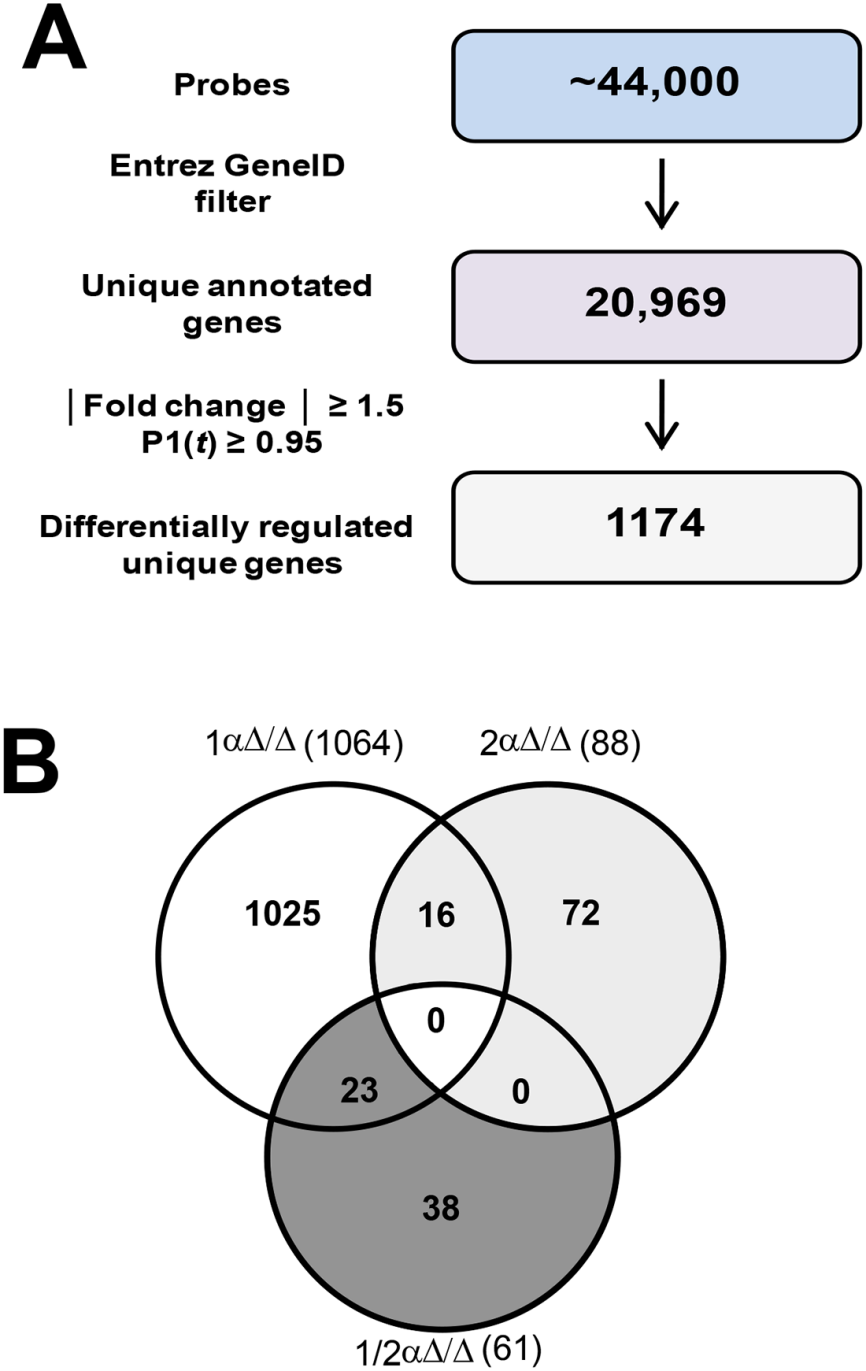
Differential gene expression in lungs of *Hif-1αΔ/Δ*, *Hif-2αΔ/Δ* and *Hif-1/2αΔ/Δ* mice. Process for identifying differentially regulated genes across all three genotypes (**A**). Comparative gene expression analysis between *Hif-1αΔ/Δ*, *Hif-2αΔ/Δ*, and *Hif-1/2αΔ/Δ* pups. The Venn diagram illustrates common and unique differentially expressed genes (**B**).

To understand the collective roles of *Hif-1α* and *Hif-2α* in the fetal lung development, gene expression was assessed using Agilent 4 × 44 K whole genome mouse microarray. The microarray contains approximately 44,000 oligonucleotide probes that represent 34,204 annotated genes including approximately 20,969 unique genes. Model-based t-values that compared treated and vehicle responses followed by Empirical Bayes analysis identified 1,174 differentially expressed genes based on a P1(t) ≥ 0.95 and absolute fold change ≥ 1.5-fold (Fig 5A). Overall, 626 genes showed an increased expression and 438genes showed decreased expression in the *Hif-1αΔ/Δ* mice as compared to control pups. The changes in gene expression in *Hif-1αΔ/Δ* mice ranged from 37-fold induction (*Slc34a1*) to 16-fold repression (*Scd1*). In *Hif-2αΔ/Δ* mice, 58genes showed increased expression and 30genes showed decreased expression, whereas in *Hif-1/2αΔ/Δ mice*, 21and 40 genes showed increased and decreased expression, respectively. The changes in gene expression ranged from 9-fold (*Fcrl6*) and 5-fold (*Gstm5*) increases to 3-fold (*Olfr583*) and 6-fold (*Apof*) decreases in *Hif-2αΔ/Δ* and *Hif-1/2αΔ/Δ* mice, respectively. Fig 5B shows how many of the genes were shared amongst the three different groups of *HIFαΔ/Δ* mice. Complete microarray gene expression data can be found in S2 Table.

### QRTPCR verification of selected microarray gene expression responses

**Fig 6:**
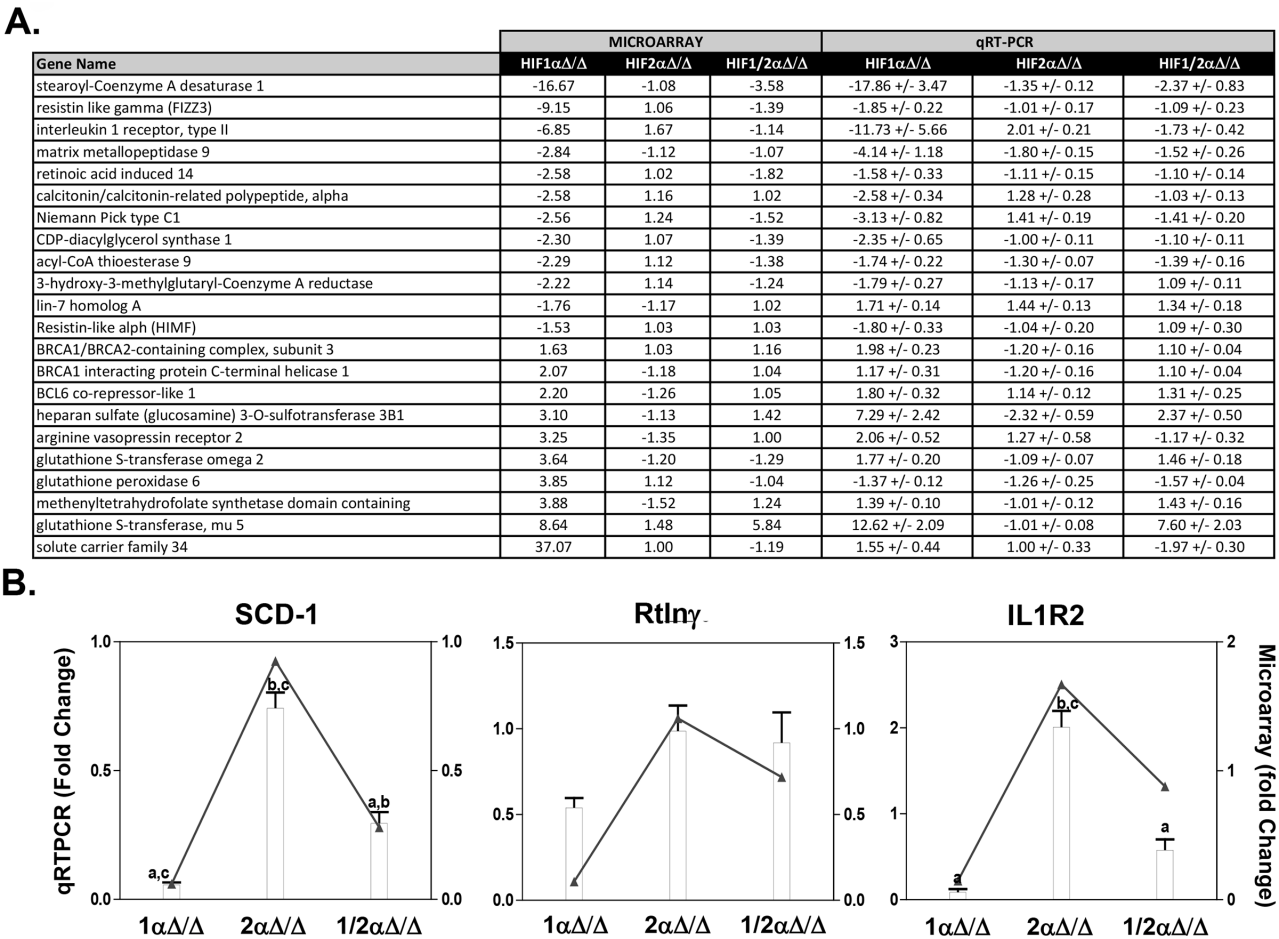
QRTPCR verification of selected microarray gene expression responses. The same RNA used for Agilent microarrays was examined by QRTPCR. All fold changes were calculated relative to controls. Fold changes of the 22 most differentially expressed genes in the microarray are compared with fold changes from qRT-PCR verification (**A**). Three represented genes shows correlation of the fold changes between microarray and qRT-PCR verification (**B**); bars (left y-axis) and lines (right y-axis) represent the fold-change values relative to *Hprt* for QRTPCR and microarray data, respectively; “a” indicates a statistical difference from *2αΔ/Δ*, “b” indicates a statistical difference from *1αΔ/Δ*, “c” indicates a statistical difference from *1/2αΔ/Δ*. Statistical significance indicates a p-value ≤ 0.05.

Quantitative real-time PCR (qRT-PCR) was used to verify the differential expression for a selected subset of differentially expressed genes representing different response profiles and functions. In general, there was a good agreement in the level of differential expression when comparing microarray and QRTPCR data (Fig 6A). Some notable genes that were verified include stearoyl-Coenzyme A desaturase 1 (*Scd1*), heparan sulfate (glucosamine) 3-O-sulfotransferase 3B1, resistin-like gamma (*Retlnγ*), and interleukin 1 receptor type II (*Il1r2*) (Fig 6B). To assess whether protein levels of SCD1 in the lung matched the pattern of gene expression, immunohistochemistry was performed (S3 Fig). In agreement with general trend of gene expression seen in Fig 6B, staining was strongly observed in lung epithelial cells of control and *Hif-2αΔ/Δ* mice, slightly less in *Hif-1/2αΔ/Δ* mice, and drastically reduced in *Hif-1αΔ/Δ* mice.

### Enrichment and Gene Network Analysis

To further elucidate the potential functional significance of these gene expression changes, enrichment and gene network analysis was performed on differentially expressed genes unique to the *Hif-1αΔ/Δ* genotype. No enrichment or gene network analysis was performed on the gene expression changes in the *Hif-2αΔ/Δ* and *Hif-1/2αΔ/Δ* mice due to the limited number of differentially expressed genes. The enrichment analysis in the *Hif-1αΔ/Δ* mice identified a number of canonical signaling pathways and gene ontology processes that were significantly enriched (FDR adjusted p-value < 0.05). These included developmental adenosine 2A receptor signaling, CFTR regulation, cellular organization, and intracellular transport (S4 and S5 Figs). When the differentially expressed genes were analyzed in the context of the standard pharmacological target classes, a significant increase in the number of kinases and a decrease in the number of receptors were observed (S3 Table). The biological significance of this observation is unclear, but a number of canonical kinase cascades are known to be regulated by or play a role in hypoxia signaling [45–47].

The enrichment analysis for the canonical signaling pathways and biological processes demonstrated considerable diversity among the pathways and processes regulated by Hif-1α. To assess the diversity of the signaling pathways involved, network topology analysis was performed on the differentially expressed genes that were unique to the *Hif-1αΔ/Δ* mice (S6 Fig). The clustering coefficient among the differentially expressed genes was 0.019 and was considerably lower than that calculated among all genes in the database (0.070). A low clustering coefficient is characterized by relatively low connectivity among the gene set and reflects the diversity in pathways involved in hypoxia signaling and lung development.

**Fig 7:**
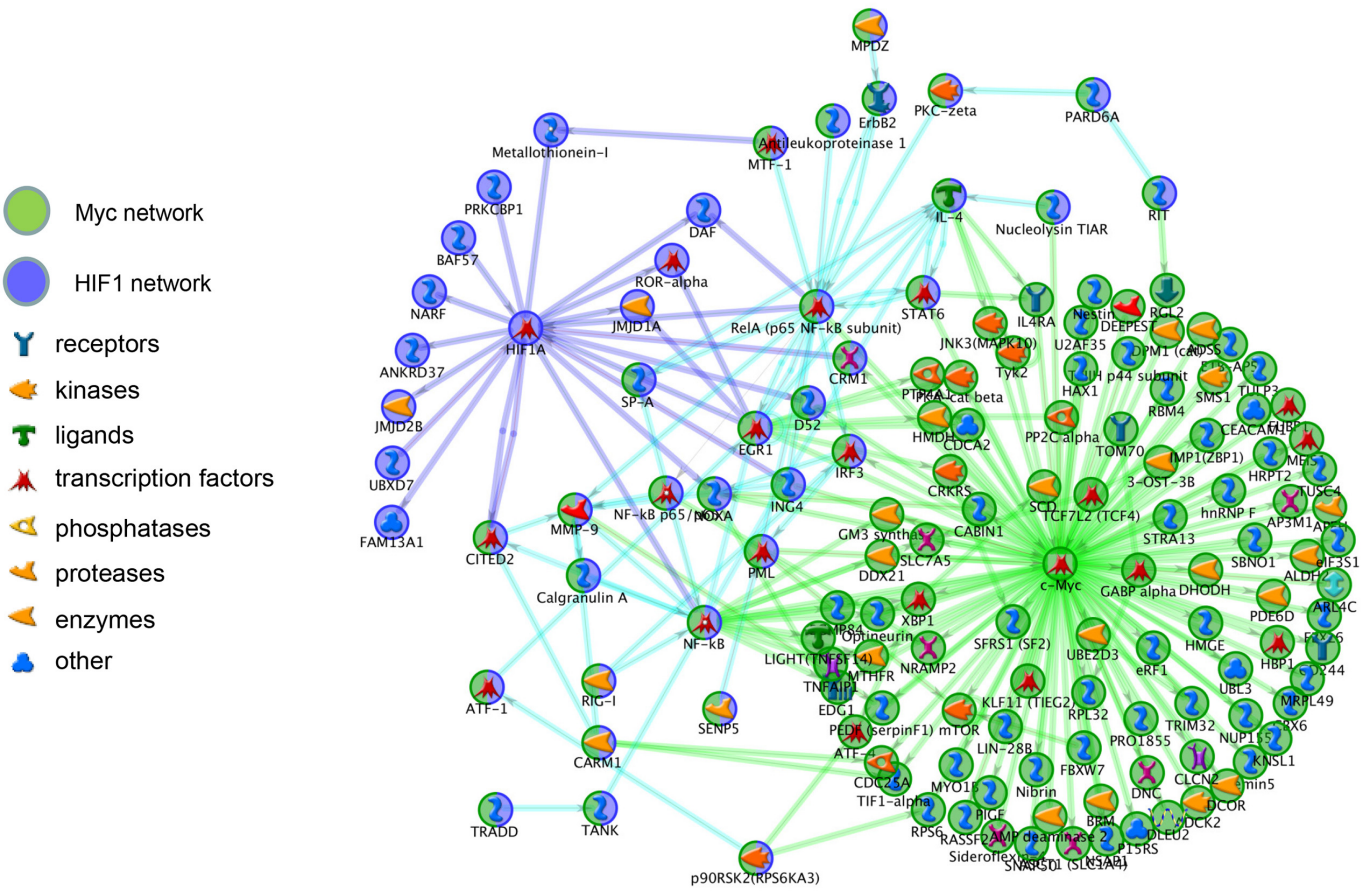
Merged Transcriptional Network with *c-Myc*. The linkage between the *Hif-1α* transcriptional network and *c-Myc* were merged and common genes in both networks were identified. *Hif-1α*-specific (blue circles), *c-Myc*-specific (green circles), and common genes (half blue, half green circles) are separated by function.

**Fig 8:**
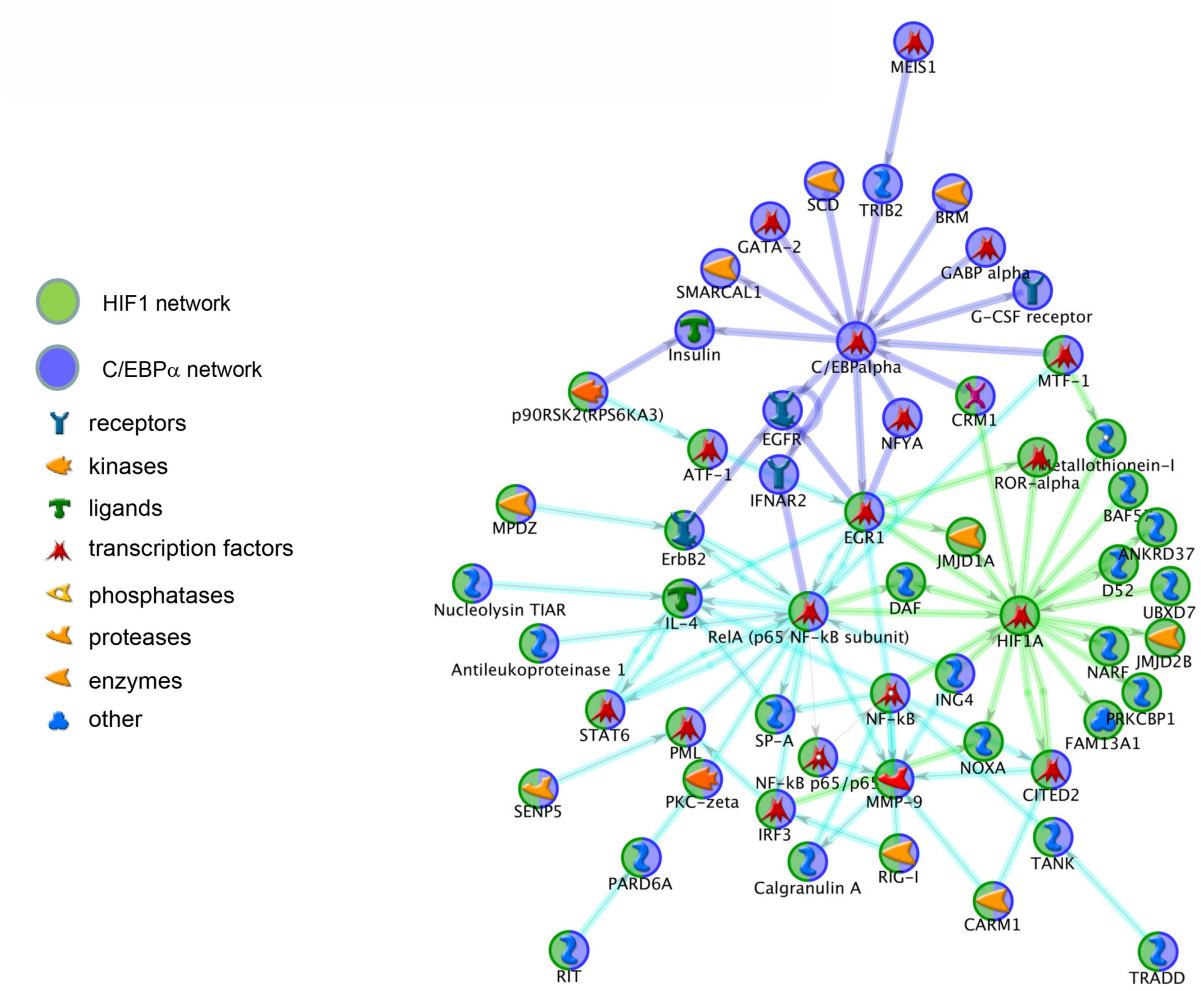
Merged Transcriptional Network with *C/ebpα*. The linkage between the *Hif-1α* transcriptional network and *C/ebpα* were merged and common genes in both networks were identified. *Hif-1α*-specific (green circles), *C/ebpα*-specific (blue circles), and common genes (half blue, half green circles) are separated by function.

To evaluate key transcription factors involved in Hif-1α signaling, a transcription regulation network was constructed for the differentially expressed genes unique to the *Hif-1αΔ/Δ mice*. Multiple transcription factors were significantly enriched within the dataset including *Hif-1α*. The linkage between the *Hif-1α* transcriptional network and other transcription factors was assessed by merging the associated network and identifying genes common to both networks. Linkages between *Hif-1α* and *c-Myc* and between *Hif-1α* and *C/ebpα* showed considerable commonality that involved genes of the *NFκB*, *ErbB2*, and *Egr-1* signaling pathways (Figs 7 and 8).

## Discussion

Normal prenatal lung development is essential for the smooth transition of the placental gas exchange to air breathing at birth. This complex process is characterized by biochemical, cellular, and ultrastructural changes that are precisely controlled by the spatiotemporal expression (or activity) of transcriptional and paracrine factors. Several transcription factors, including *Klf5*, *Foxa2*, *C/ebpα*, *Gata-6*, *β-catenin*, and *Nkx2*.*1* (also known as*Ttf-1*) have already been added to the list of indispensable genes for lung development [4, 48–53]. The current challenge to clarifying these and other transcriptional programs in lung development is to understand the networking between each and the overlapping roles they may play. A revealing recent study by Xu *et al*. analyzed a time course of genome-wide microarray data from E15 to birth of two different mouse strains highlighting not only timing of major transcriptional programs, but also that strain and gestational age play vital roles in this programming [3]. This approach highlights the importance of programming networks over individual factors and the interrelationships of the various transcriptional players. Further testing for other developmentally critical transcriptional factors and their wider roles in more complex networks is required to provide a more comprehensive understanding of the signaling cascades involved in lung development. This understanding is crucial for optimizing new therapies for the many lung disorders, which have perturbations in developmental signaling pathways.

Recently our laboratory used mice with the *SPC-rtTA/tetO7-Cre* system to drive *Hif-1α* deletion in lung epithelial cells from E4.5 to birth, which resulted in compromised lung development and altered surfactant protein metabolism. The epithelial-specific loss of *Hif-1α* leads to neonatal RDS due to impaired alveolar epithelial differentiation. Morphogenesis of conducting airways, however, was unaffected suggesting the involvement of *Hif-1α* in late events in lung development, including cellular differentiation, rather than in the early lung biogenesis and airway branching morphogenesis [30]. In contrast to *Hif-1α* deletion, a recent study by Bridges *et al*. used the *SPC-rtTA/(tetO)*_*7*_ model to drive induction of two different stable *Hif-1α* constructs from E6.5 onward. Mice with the first transgenic construct, a *ΔODD-N803A* mutant, were phenotypically similar to our *Hif-1αΔ/Δ* mice with no observable defects in branching morphogenesis but profound alveolar epithelial immaturity. In contrast, mice receiving the other stable *Hif-1α* construct, a triple point mutation (*Hif-1α-TPM*) to alanine of prolines 402 and 564 and asparagine 803 showed disruption in branching morphogenesis, increased epithelial glycogen stores, marked lymphangiogenesis, and induction of several *Hif-1α*-responsive genes in the lungs. Interestingly, the *ΔODD-N803A* construct inhibited endogenous *Hif-1α* stability in HeLa cells and had much lower activity in HRE-luciferase reporter assays than the *Hif-1α-TPM* construct; the authors suggested that perhaps this endogenous *Hif-1α* disruption was responsible for the phenotypic differences between the *ΔODD-N803A* and *Hif-1α-TPM* mice and, perhaps, the similarities to the *Hif-1αΔ/Δ* mice. They also showed that *Cdkn1a* (*p21*) was induced in *Hif-1α-TPM* mice and not in *ΔODD-N803A* mice, which correlated with decreased epithelial cell proliferation in *Hif-1α-TPM* mice [27]. Previous work has shown the importance of *Hif-1α* in displacing *c-Myc* repressor from the *p21* promoter [54]. The robust connectivity between *Hif-1α* and the *c-Myc* networks in our data support the importance of these networks in lung development and, more specifically, for branching morphogenesis.

Interestingly, Tibboel and colleagues created a mouse constitutively expressing a *Hif-1α* transgene with ODD deletion [31]. These mice, in stark contrast to results of Bridges et al., did not reveal developmental abnormalities, but had increased alveolarization and vascularization. One possibility for these differences is the presence of endogenous N803, outside the ODD, that could allow factor inhibiting HIF (FIH) to downregulate expression in this construct in specific ways that differ from the *Hif-1αΔODD-N803A* or triple point mutant (*P402A*, *P564A*, *N803A*). Additionally, interaction of CBP/p300 with activated HIF-1α/ARNT heterodimer may affect other important gene expression pathways that change the phenotypic outcome.

Although *Hif-1*α is the most ubiquitously expressed HIFα isoform, *Hif-2*α has unique expression patterns and immunohistochemistry suggesting non-overlapping roles of *Hif-2α* and *Hif-1α* in lung development. Here, our results suggest that lung-specific loss of *Hif-2α* does not result in respiratory distress. This most likely stems from near complete loss of *Hif-2α* from every cell type of the lung in the current model (based on early expression of *Sp-c* in lung development [35]), and genotype and/or strain differences of the mice used [17]. Previous reports using traditional *Hif-2α* knockout mice generated in a specific genetic background drew conclusions based on a percentage of mice that survived until parturition. Differences in genetic background, genotype, and the loss of *Hif-2α* from other organs, most notably the cardiac tissue, are potentially confounding factors. The data presented here suggests that the specific loss of *Hif-2α* from the lung epithelium leads to no overt phenotype and may lead to more developed lungs compared to genotype controls (Fig 3). Huang and colleagues used the *SPC-rtTA/tetO7* system to express a stable *Hif-2α* isoform (containing *P531A* and *N847A* mutations) from E6.5 onward. Mice with stable *Hif-2α* expression displayed respiratory distress and died within 6 hours of parturition. Histological and ultrastructural analyses showed increased alveolar size with apparent decreases in alveolar type I (ATI) cell markers and alveolar type II (ATII) cells lacking formed lamellar bodies with increased glycogen stores [32]. Clearly the overexpression of *Hif-2α* in this cell population affects alveolarization and seems to inhibit type I pneumocyte differentiation and septal development. It is interesting to note that these data collectively suggest *Hif-2α* expression is detrimental to lung development, yet *Hif-2α* expression from many studies is seen to progressively increase toward parturition and maturing lung development. Several possibilities exist to explain these discrepancies, but more careful time-course analyses of our *Hif-2αΔ/Δ* mice, beyond the scope of this study, are necessary before meaningful comparisons of *Hif-2α* in lung development can be made.

The results suggest that HIFs participate in a transcription factor network that is required for proper lung development. Data from *Hif-1αΔ/Δ* mice suggest that HIF-1 participates downstream of key factors, such as *C/ebpα*, *Foxa2*, and *Nkx2*.*1*, and upstream of other factors, such as *Hif-2α*, *β-catenin*, resistin like γ (*Retnlγ*), and interleukin 1 receptor type II (*Il1R2*). *Retnlγ*, also known as *Fizz3*, is significantly down-regulated in *Hif-1α*-deficient lungs but rescued in *Hif-1/2αΔ/Δ* mice. *Retnlγ* is a member of family of hormones that are derived from adipose tissue. In mice, there are four known members, resistin, and three resisitin-like molecules (*Retnl α*, *β*, *and γ*). These hormones have varied tissue expression profiles and the tissue-specific functions have not been completely elucidated. The resistin family has a demonstrated role in lung function. *Retnlα* (aka *Fizz1* and *Himf*) was increased in the lavage fluid of mice following induction of an asthma-like phenotype through ovalbumin challenge and plays an important role in the early airway remodeling in this model [55]. Additionally, *Retnlα* ^*-/-*^ mice showed exacerbated Th2 responses in helminth models that was reversible with administration of recombinant *Retnlα* [56]. Expression of the human isoform, *RELMα*, is increased in patients suffering from scleroderma [57]. *Retnlβ* is most closely associated with a Th2 inflammatory response in the lung and is an “asthma signature” gene. Direct delivery of *Retnlβ* into the lung of mice increases macrophage accumulation, goblet cell hyperplasia, and perivascular and peribronchial collagen deposition. *Retnlβ* deficient mice have a compromised ability to induce collagen and goblet cell hyperplasia following Aspergillus challenge [58]. *Retnlγ*, the fourth member of the family was found to be highly expressed in the lung where its expression is significantly decreased by exposure to cigarette smoke [59]. Recently, postnatal loss (PN4-30) of *Hif-1α* from Clara and ATII cells was shown to bias the tissue towards a Th2 inflammation in a cobalt-induced toxicity model, similar to asthma [60]. Further studies in an ovalbumin sensitization/challenge model shows that PN4-14*Hif-1αΔ/Δ* mice have an exacerbated eosinophilic response with increased total lung resistance, which is not seen in adult mice that lost *Hif-1α* from PN32-42 [61]. It is interesting to speculate that HIF-mediated regulation of the resistin family of hormones during lung development might play a role in establishing this change in inflammatory response.

Interleukins are a family of cytokines that regulate a host of inflammatory responses. There are two Interleukin 1 (IL1) proteins, IL1α and IL1β. These molecules are pro-inflammatory cytokines that regulate leukocyte migration, induce fevers, and many other functions. These molecules are also important developmental regulators. For example, intra-amniotic delivery of IL1α promotes lung maturation [62]. Moreover, exogenous delivery of IL1α or IL1β can alter surfactant protein expression; however, these changes are dependent upon the developmental stage in which the interleukin is delivered [62]. These results suggest that proper IL1 levels are critically important for lung development. Both IL1 isoforms interact with one of two types of IL1 receptors. The type I receptor (IL1R1) is the canonical transmembrane receptor that initiates intracellular signaling cascades upon ligand binding. The type II isoform of the IL1 receptor (IL1R2) lack the cytoplasmic domain of IL1R1 and is therefore signaling deficient. IL1R2 can also be released from the plasma membrane by the action of matrix metalloproteinases. IL1R2 acts as a decoy receptor for circulating IL1α and IL1β preventing their ability to activate the IL1R1. Given that IL1α and IL1β levels can alter the developmental program of the lung and IL1R2 can modify the activity of circulating IL1α and IL1β, proper Il1R2 levels might be important for proper development of the lung. *Il1r2* was significantly down-regulated in the *Hif-1αΔ/Δ* pups and restored in the *Hif-1/2αΔ/Δ* mice (Fig 8). These results suggest that HIF-mediated changes in *Il1r2* and the subsequent alterations in active IL1 might explain the changes observed in the development of the lungs.

The integration of the *Hif-1α* network and that of *C/ebpα* and *c-Myc* overlapped at *Nf-κb*. Microarray analysis showed a significant down-regulation of *p65/RelA* in the *Hif-1αΔ/Δ* mice that was rescued in the *Hif-1/2αΔ/Δ* mice. Recently, over-expression of *p65/RelA* from an *Sp-c* promoter was demonstrated to influence the differentiation of alveolar Type I and II cells [63]. Moreover, high levels of RELA have been observed in distinct regions of the lung mesenchyme during development and altering this expression impacted budding in vitro [64]. Finally, *Nf-κb* has a demonstrated role in various lung diseases, including asthma, chronic obstructive pulmonary disease (COPD), and acute lung toxicity. The identification of *RelA* as a *Hif-1α*-modulated gene suggests that *RelA* plays an important role in the final stages of lung development.

In conclusion, the present study provides evidence that *Hif-1α* is centrally located in the transcriptional network necessary for proper lung development. Lung-specific deletion of *Hif-2α* alone, however, is not lethal to the neonatal pups and there was no evidence of pathology. Interestingly, the rescue of respiratory distress was observed after simultaneous deletion of both *Hif-1α* and *Hif-2α*. Microarray analysis identified several genes associated with lung development that are differentially regulated, including *Retlnγ* and *Il1r2*. Finally, pathway and network analysis suggests that changes in these critical factors are due, in part, to changes in *C/ebpα*, *c-Myc*, and *Nf-κb* signal transduction. Further exploration of the relationship between HIF and the effects these changes have on downstream modulators of lung development, such as *Il1r2* and *Retnlγ*, may further elucidate the etiology of RDS and the signals necessary for proper lung development.

## Supporting Information

S1 ARRIVE Checklist ARRIVE Guidelines Checklist. (PDF)

S1 Fig HIF-1α Immunohistochemistry. Lung sections from control (no DOXY, **A, C, E**) and*Hif-1αΔ/Δ* (**B**), *Hif-2αΔ/Δ* (**D**), and *Hif-1/2αΔ/Δ* (**F**) pups were immunostained for HIF-1α as described in materials and methods. Representative positively stained cells for HIF-1α are depicted by solid arrows. AD = alveolar duct, a = alveolus.
(TIF)

S2 Fig HIF-α Immunohistochemistry. Lung sections from control (**A, C, E**) and *Hif-1αΔ/Δ* (**B**), *Hif-2αΔ/Δ* (**D**), *Hif-1/2αΔ/Δ* (**F**) pups were immunostained for HIF-2α as described in materials and methods. Positively stained cells for HIF-2α are depicted by solid arrows. AD = alveolar duct, a = alveolus.
(TIF)

S3 Fig IHC for SCD1. Neonatal lungs were immunostained for SCD1 as described in Materials and Methods. Control mice (NO DOX *Hif-1α* ^*fl/fl*^) are shown in (**A**), *Hif-1αΔ/Δ* in (**B**), *Hif-2αΔ/Δ* in (**C**) and *Hif-1/2αΔ/Δ* in (**D**).
(TIF)

S4 Fig Enrichment Analysis by GeneGo. Genes that were differentially regulated in the *Hif-1αΔ/Δ* mice as compared to control (ctrl) animals (P1(t) ≥ 0.95) were analyzed for enriched GeneGo pathways. The top 15 out of 24 enriched GeneGo pathways and their respective FDR adjusted p-values are listed.
(TIF)

S5 Fig Enrichment Analysis by Gene Ontology (GO). Genes that were differentially regulated in the *Hif-1αΔ/Δ* mice as compared to control (ctrl) animals (P1(t) ≥ 0.95) were analyzed for enriched Gene Ontology (GO) pathways. The top 20 out of 112 GO processes and their respective FDR adjusted p-values are listed.
(TIF)

S6 Fig Top 10 networks constructed using the genes significantly altered by *in utero* deletion of Hif-1α from lung epithelium. (TIF)

S1 Table Primer List for qRT-PCR. (TIF)

S2 Table Complete List of Differentially Regulated Genes in Lungs of *Hif-1αΔ/Δ* Mice. (TIF)

S3 Table Enrichment analysis among broad protein classes for gene expression changes unique to HIF-1 genotype. (TIF)
