# Supplementary figures and images for: Loss of Hif-2α Rescues the Hif-1α Deletion Phenotype of Neonatal Respiratory Distress In Mice
Source: PLoS One. 2015 Sep 30;10(9):e0139270. doi: 10.1371/journal.pone.0139270 (PMC4589293; doi:10.1371/journal.pone.0139270)

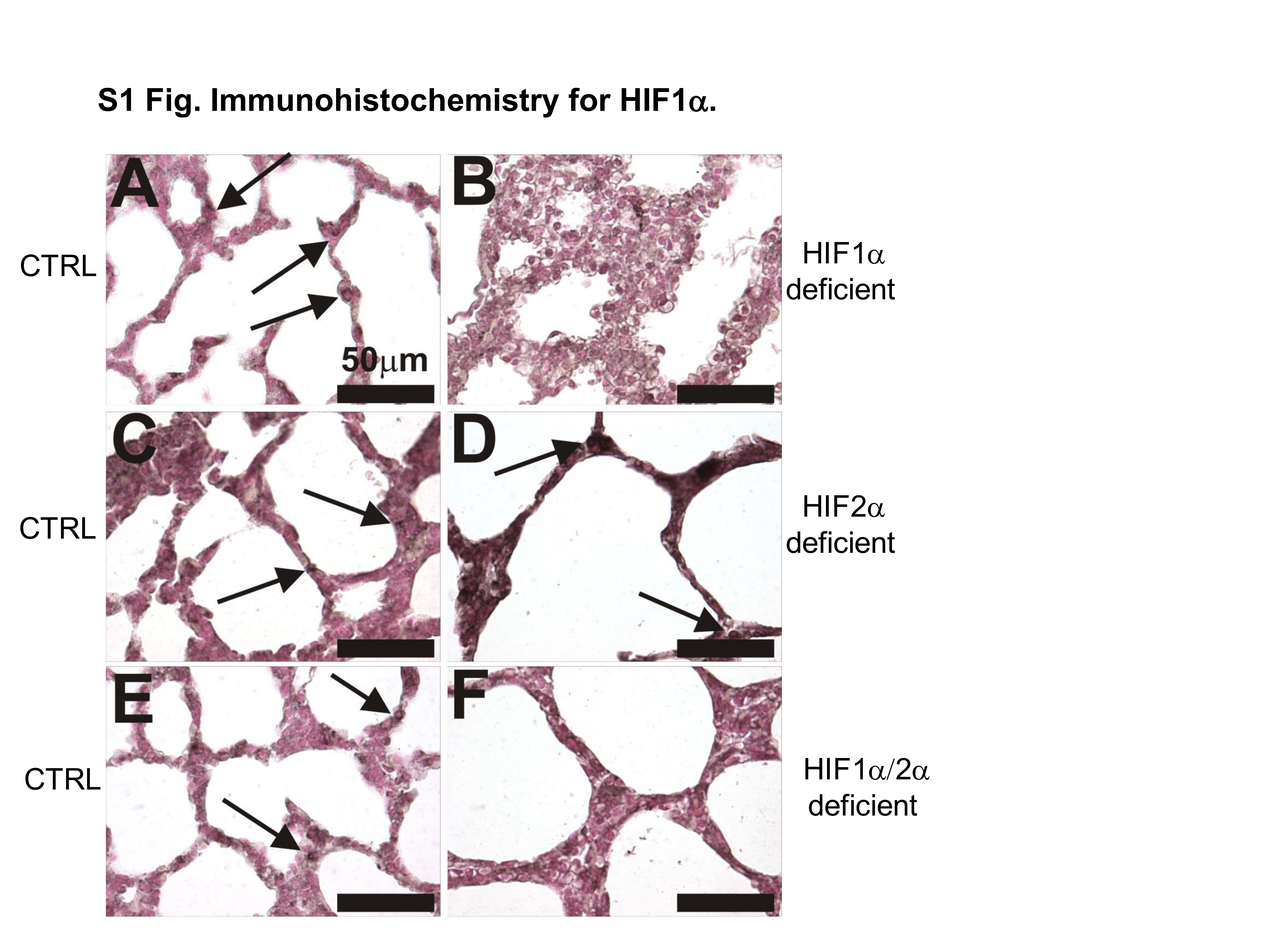

Supplement: S1 Fig — Lung sections from control (no DOXY, A, C, E) andHif-1αΔ/Δ (B), Hif-2αΔ/Δ (D), and Hif-1/2αΔ/Δ (F) pups were immunostained for HIF-1α as described in materials and methods. Representative positively stained cells for HIF-1α are depicted by solid arrows. AD = alveolar duct, a = alveolus. (TIF) [file pone.0139270.s002.tif]

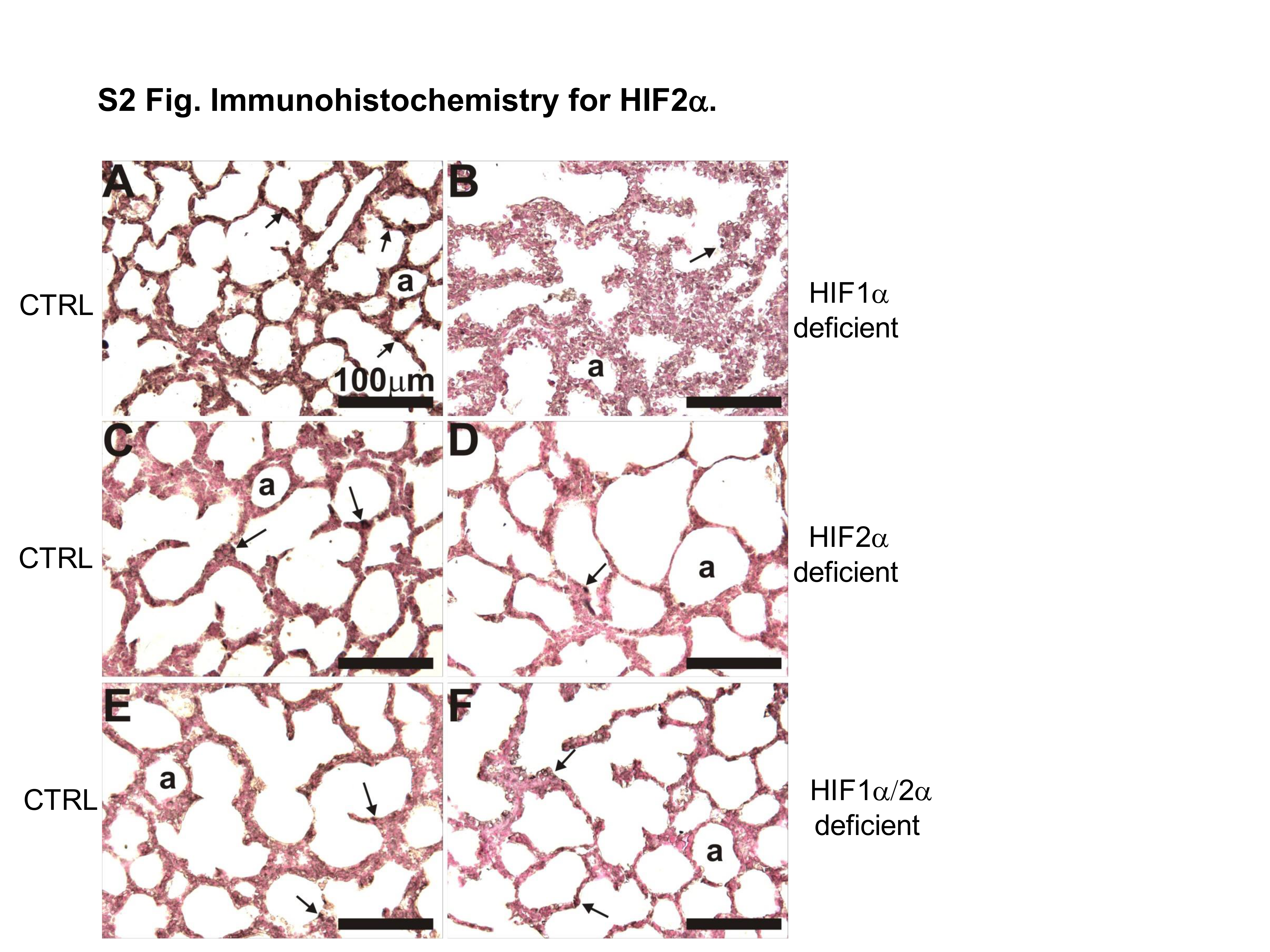

Supplement: S2 Fig — Lung sections from control (A, C, E) and Hif-1αΔ/Δ (B), Hif-2αΔ/Δ (D), Hif-1/2αΔ/Δ (F) pups were immunostained for HIF-2α as described in materials and methods. Positively stained cells for HIF-2α are depicted by solid arrows. AD = alveolar duct, a = alveolus. (TIF) [file pone.0139270.s003.tif]

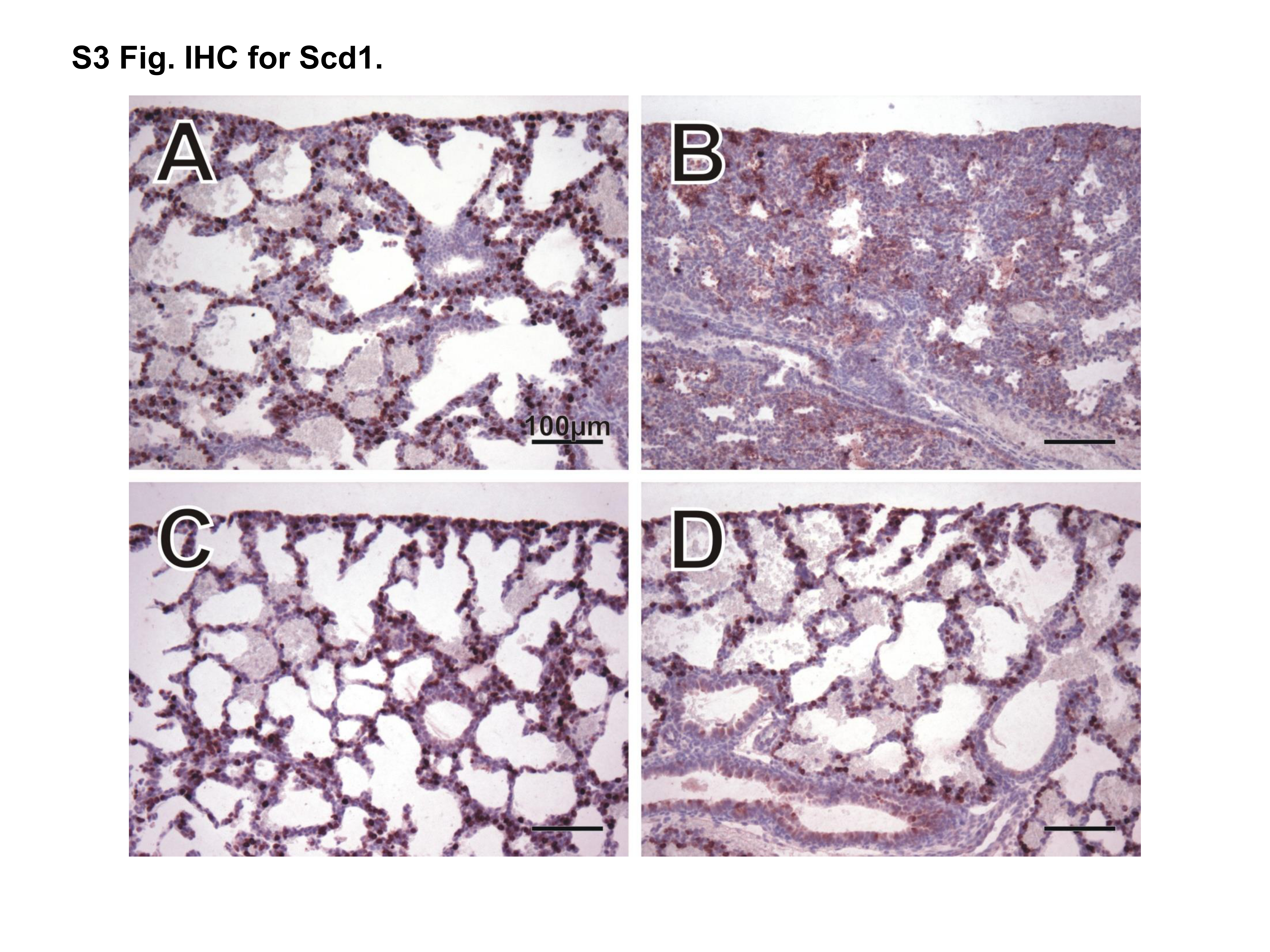

Supplement: S3 Fig — Neonatal lungs were immunostained for SCD1 as described in Materials and Methods. Control mice (NO DOX Hif-1α fl/fl) are shown in (A), Hif-1αΔ/Δ in (B), Hif-2αΔ/Δ in (C) and Hif-1/2αΔ/Δ in (D). (TIF) [file pone.0139270.s004.tif]

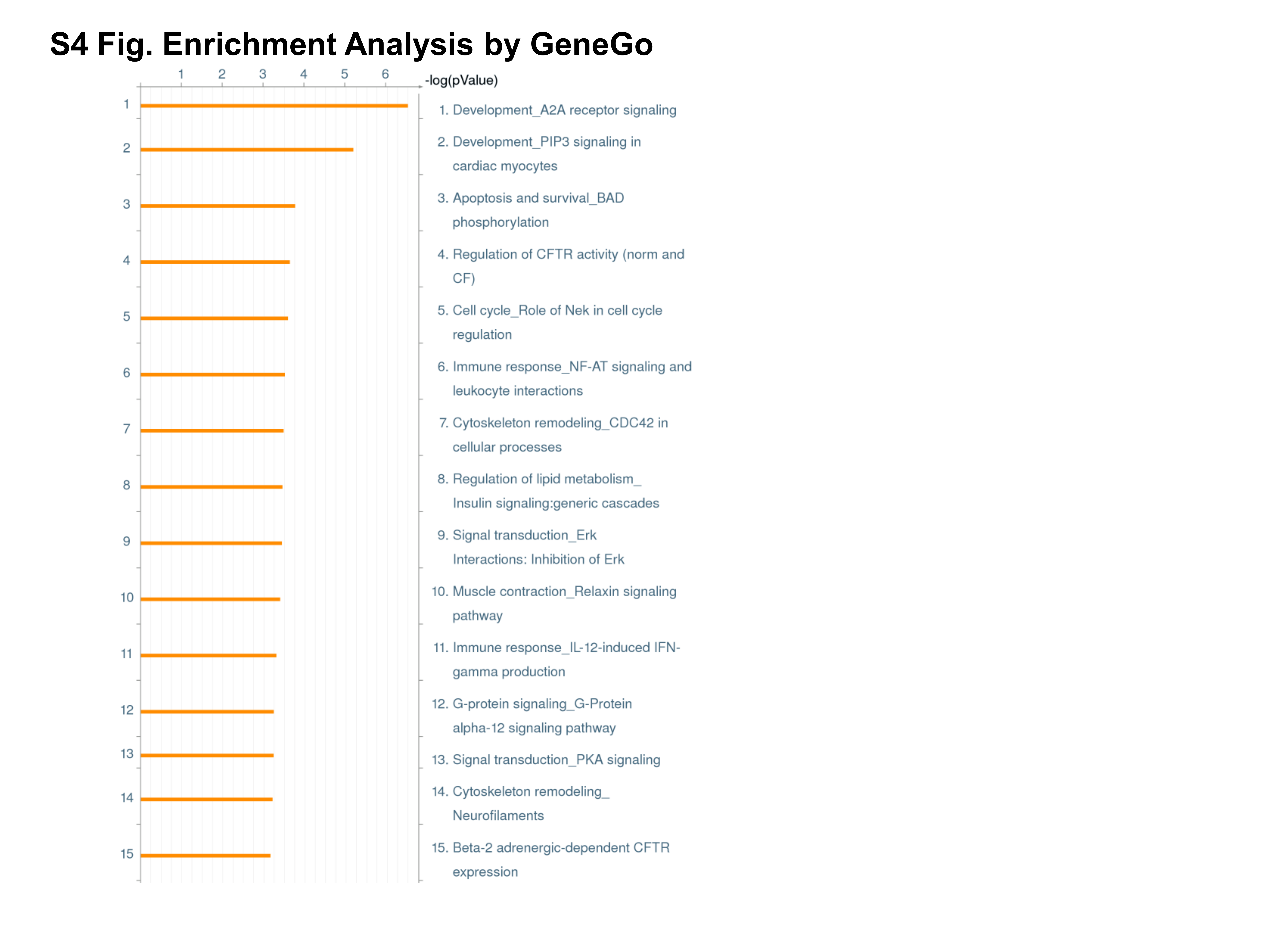

Supplement: S4 Fig — Genes that were differentially regulated in the Hif-1αΔ/Δ mice as compared to control (ctrl) animals (P1(t) ≥ 0.95) were analyzed for enriched GeneGo pathways. The top 15 out of 24 enriched GeneGo pathways and their respective FDR adjusted p-values are listed. (TIF) [file pone.0139270.s005.tif]

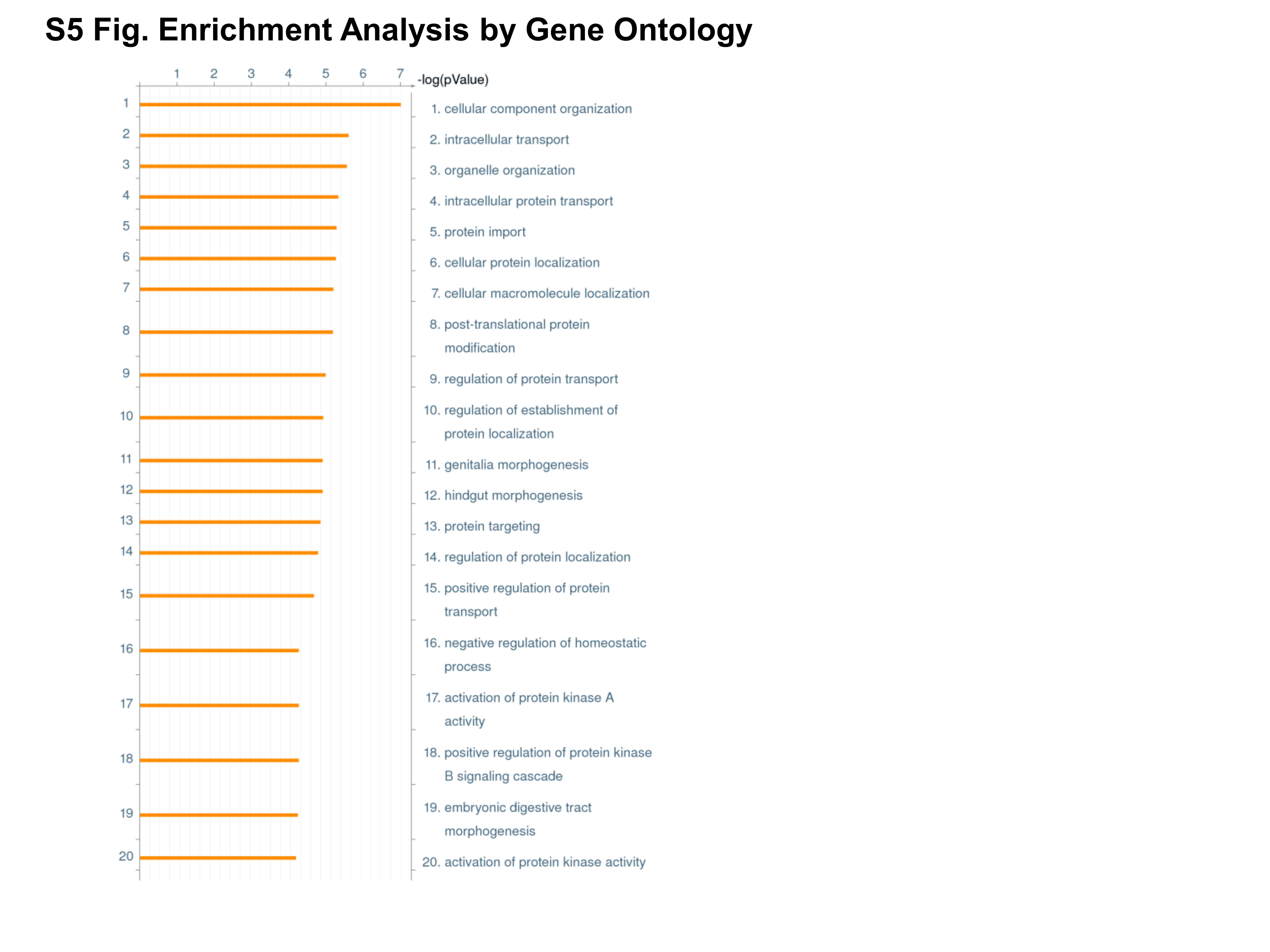

Supplement: S5 Fig — Genes that were differentially regulated in the Hif-1αΔ/Δ mice as compared to control (ctrl) animals (P1(t) ≥ 0.95) were analyzed for enriched Gene Ontology (GO) pathways. The top 20 out of 112 GO processes and their respective FDR adjusted p-values are listed. (TIF) [file pone.0139270.s006.tif]

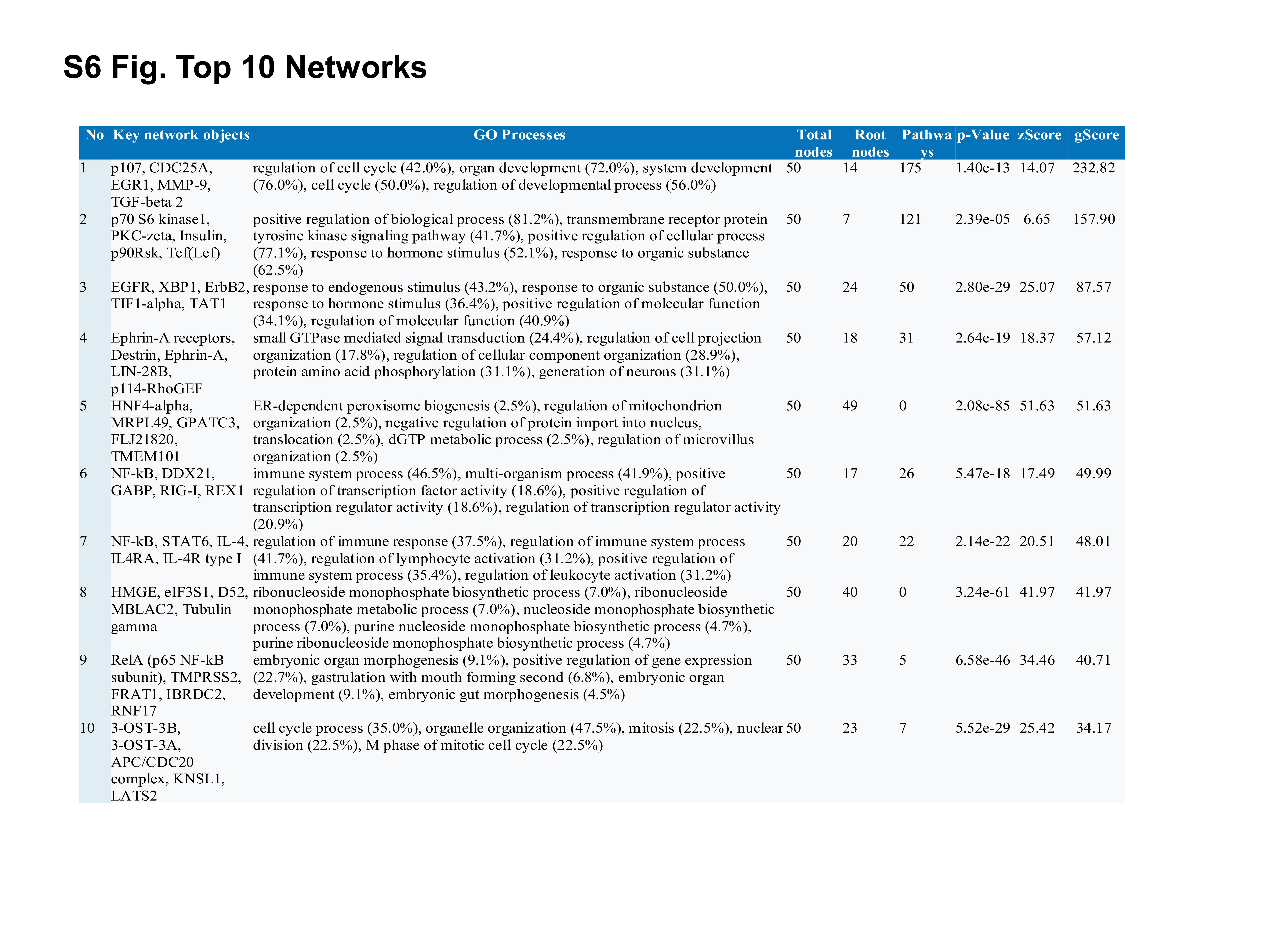

Supplement: S6 Fig — (TIF) [file pone.0139270.s007.tif]

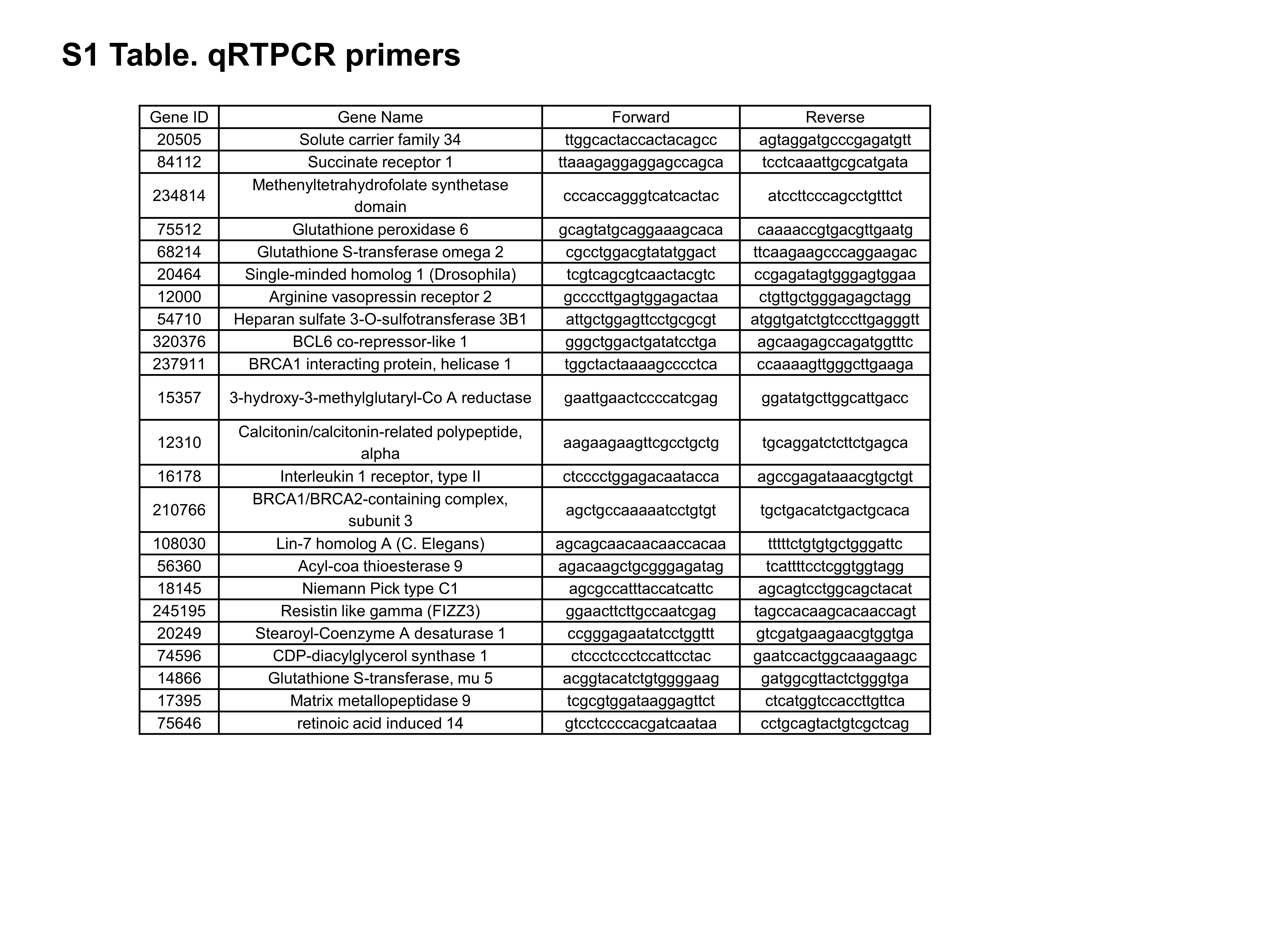

Supplement: S1 Table — (TIF) [file pone.0139270.s008.tif]

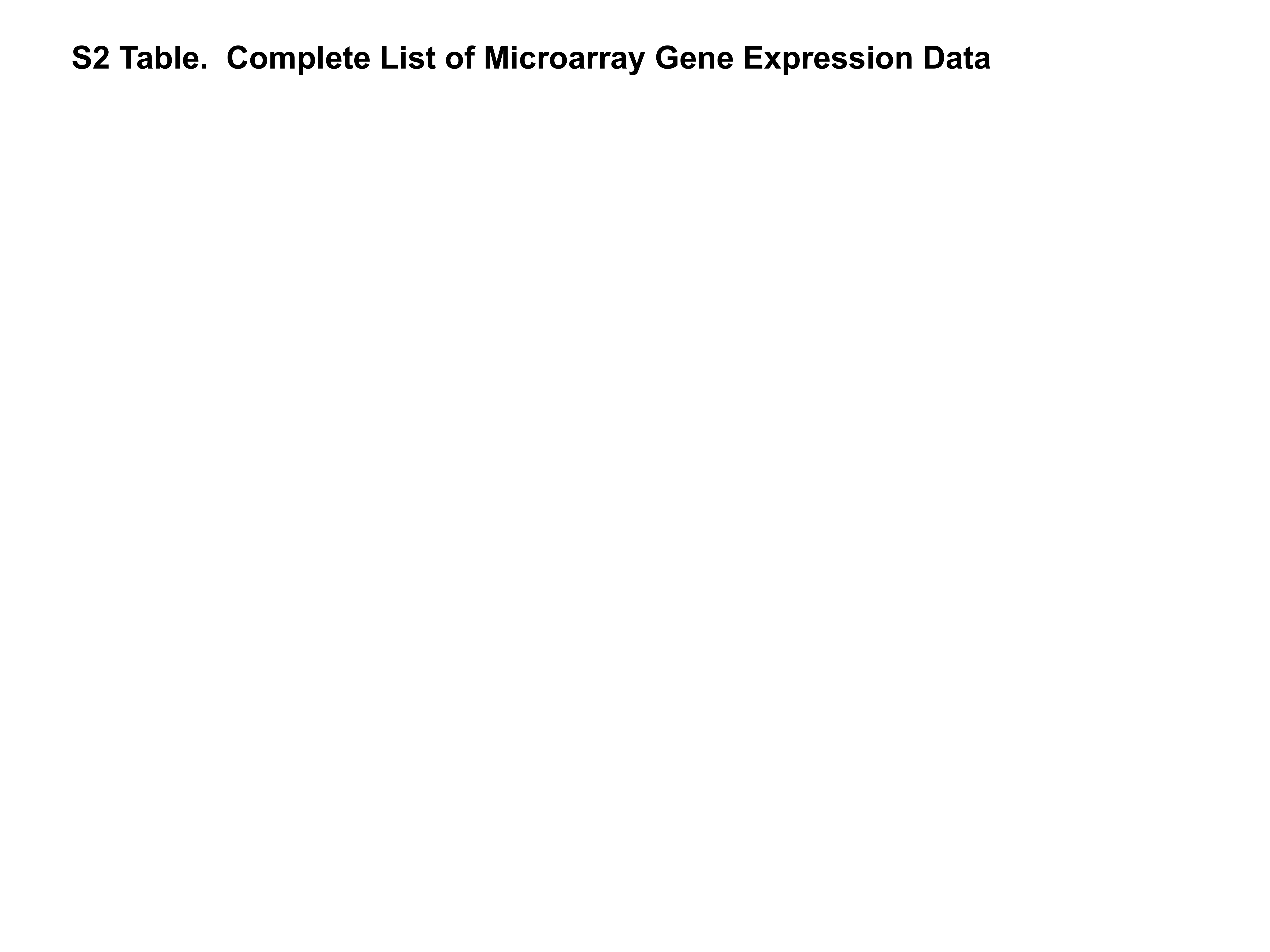

Supplement: S2 Table — (TIF) [file pone.0139270.s009.tif]

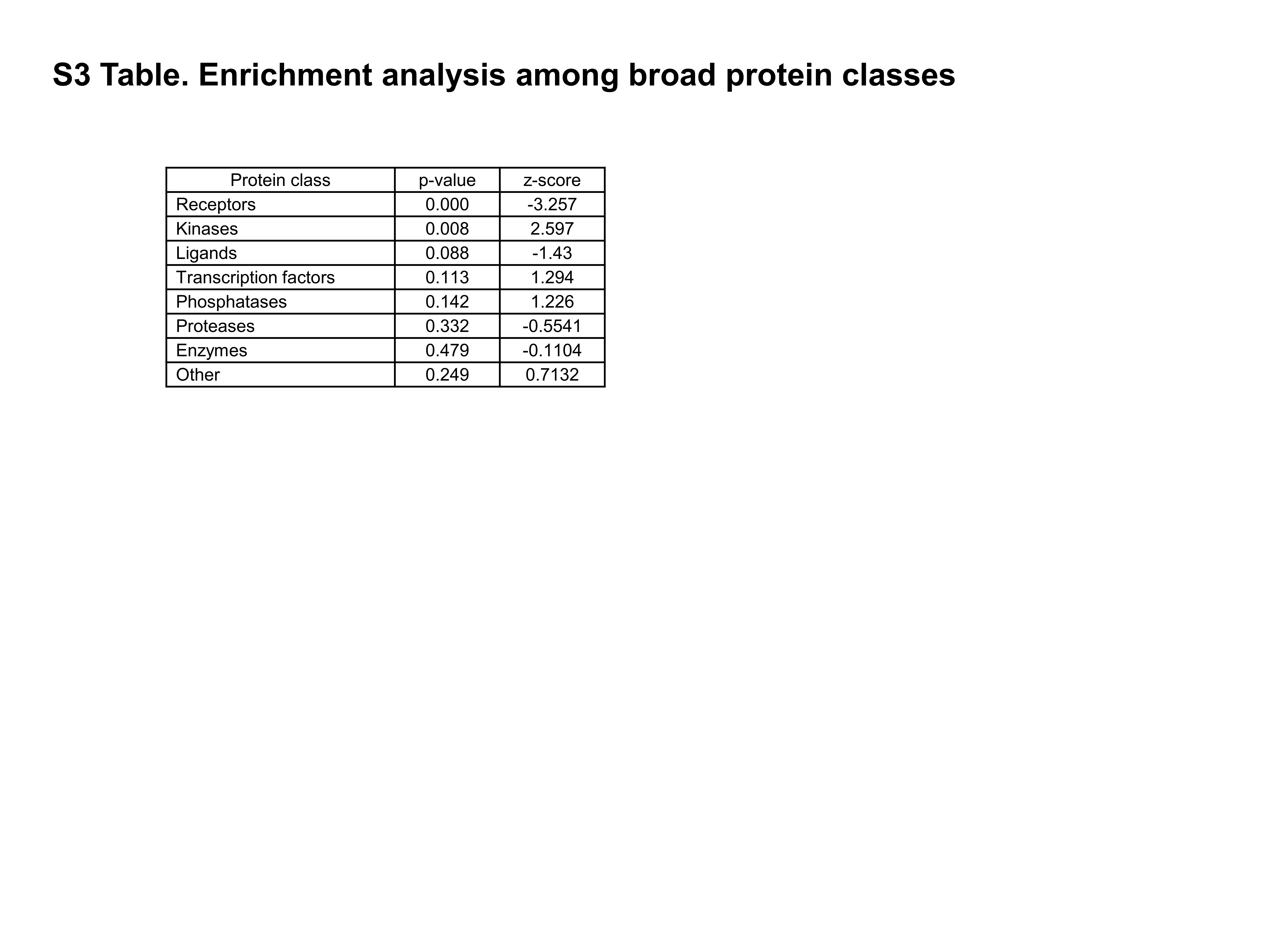

Supplement: S3 Table — (TIF) [file pone.0139270.s010.tif]
